# Impact of Ancestral Differences and Reassessment of the Classification of Previously Reported Pathogenic Variants in Patients With Brugada Syndrome in the Genomic Era: A SADS-TW BrS Registry

**DOI:** 10.3389/fgene.2018.00680

**Published:** 2019-01-04

**Authors:** Ching-Yu Julius Chen, Tzu-Pin Lu, Lian-Yu Lin, Yen-Bin Liu, Li-Ting Ho, Hui-Chun Huang, Ling-Ping Lai, Juey-Jen Hwang, Shih-Fan Sherri Yeh, Cho-Kai Wu, Jyh-Ming Jimmy Juang, Charles Antzelevitch

**Affiliations:** ^1^Department of Internal Medicine, Cardiovascular Center and Division of Cardiology, National Taiwan University Hospital, Taipei, Taiwan; ^2^Department of Public Health, Institute of Epidemiology and Preventive Medicine, National Taiwan University, Taipei, Taiwan; ^3^Department of Environmental and Occupational Medicine, National Taiwan University Hospital, Taipei, Taiwan; ^4^Lankenau Institute for Medical Research, Wynnewood, PA, United States; ^5^Lankenau Heart Institute, Wynnewood, PA, United States; ^6^Sidney Kimmel Medical College, Thomas Jefferson University, Philadelphia, PA, United States

**Keywords:** Brugada syndrome, sudden cardiac death, inherited cardiac arrhythmia syndrome, allele frequency, pathogenic variants

## Abstract

Brugada syndrome (BrS) is a heritable disease that results in sudden cardiac death. In the exome/genomic era, certain reported pathogenic variants in some genetic diseases have been reclassified as benign owing to their high frequency in some ancestries. In the present study, we comprehensively reassessed all previously reported pathogenic variants of BrS. We collected all pathogenic variants of BrS reported in the Human Gene Mutation Database and ClinVar throughout April 2017. We compared the minor allele frequency (MAF) of each variant among different ancestries by searching public whole-genome and exome databases. After considering the maximum credible allele frequency, variants with a MAF ≥ 0.001 were considered to be of questionable pathogenicity. We also investigated the percentage of SCN5A variants with a MAF ≥ 0.001 in 124 BrS patients from the Han Chinese population. We collected a total of 440 BrS variants, of which 18 had a MAF ≥ 0.001. There was a greater percentage of non-SCN5A variants with a MAF ≥ 0.001 than of SCN5A variants (21.8 versus 1.6%, *p* < 0.0001). There were fewer frameshift and nonsense mutations than missense mutations (0.9 versus 5.6%, *p* = 0.032). Of the 18 variants, 14 (77.8%) were present only in the reference Asian population. In our cohort, we identified two SCN5A variants (p.A226V and p.V1340I) with MAFs ≥ 0.001 (0.45%). In conclusion, ancestral differences are important when considering the pathogenicity of BrS variants, especially in the case of missense variants and non-*SCN5A* variants, which may be pathogenic in some ancestries but only disease-predisposing in others.

## Introduction

Brugada syndrome (BrS) – a heritable arrhythmic disease responsible for sudden cardiac death (SCD) in patients with structurally normal hearts – was first reported by Brugada and Brugada (1992). It accounts for 4% of all sudden deaths and up to 20% of sudden deaths in patients without structural cardiac disease (Antzelevitch et al., 2005b), and has been identified as the same entity previously designated as sudden unexpected nocturnal death syndrome (SUNDS) (Vatta et al., 2002). Moreover, the prevalence of BrS is highest in the Asian population (Juang et al., 2015), which may be attributed to a distinct genetic background.

Numerous genes are associated with BrS, including *SCN5A*, *SCN10A*, *SCN1B*, *SCN2B*, *SCN3B*, *KCNH2*, *KCND2*, *KCND3*, *KCNE3*, *KCNE5*, *KCNJ8*, *CACNA1C*, *CACNA2D1*, *CACNB2*, *ABCC9*, *HCN4*, *PKP2*, *SLMAP*, *TRPM4*, *RANGRF*, *GPD1L*, *FGF12*, and *SEMA3A* (Fernandez-Falgueras et al., 2017); most encode proteins that control the transmembrane ion currents responsible for electrical impulses. The identification of pathogenic variants is crucial because it can help further investigation of the disease mechanism and facilitate family screening. To determine the pathogenicity of a variant, the American College of Medical Genetics and Genomics (ACMG) suggests taking into consideration the population allele frequency, the effect on protein structure, and the results of functional studies (Richards et al., 2015).

The expected allele frequency of a causative variant is related to the disease prevalence, the penetrance, and to the genetic contribution made by the causative variant to the disease (Whiffin et al., 2017). In other words, the prevalence of a monogenic disease is the summation of the contributions of all the pathogenic variants in a population. The expected allele frequency of each variant can vary across populations, depending on its contribution to the monogenic disease. Therefore, the pathogenicity of a variant with an allele frequency much higher than expected should be investigated carefully. The first universal genome-wide study regarding allele frequency was the 1000 Genomes Project (1000G). As the sample size increased in the Exome Aggregation Consortium (ExAC) and then the Genome Aggregation Database (gnomAD), it was expected that these studies would become more representative of the true population frequency. Ancestral differences in numerous variants were revealed, and some previously reported pathogenic variants have been reclassified as benign or disease-predisposing owing to their high frequencies in some ancestries (Manrai et al., 2016; Clemens et al., 2018). Although there have been functional *in vitro* studies of some of these variants, they could be considered disease-predisposing in high-frequency ancestries because the polymorphism of genes among ancestries is complex and may have protective effects.

Therefore, a local reference for population frequency is invaluable in determining the pathogenicity of a variant in a given ancestry. Current large-scale ancestry-specific databases for population frequency include the Taiwan Biobank (TWB), the integrative Japanese Genome Variation Database (iJGVD), and the NHLBI Grand Opportunity Exome Sequencing Project (ESP6500) for European Americans (EAs) and African Americans (AAs).

The present study was performed to investigate the ancestral differences in previously reported pathogenic variants of BrS, with the expectation that the pathogenicity of variants with relatively high allele frequencies in some ancestries should be reassessed. Because the prevalence of BrS is highest in the Asian population, we used our BrS cohort (TW-BrS registry) to validate the clinical impact of these variants, which were listed as disease-causing or pathogenic variants in the Human Gene Mutation Database (HGMD) and ClinVar.

## Materials and Methods

### Acquisition of Reported Pathogenic Variants

We collected the pathogenic and likely pathogenic variants of BrS reported in ClinVar^1^ (Landrum et al., 2016), and the disease-causing mutations in HGMD - Professional^2^ (Stenson et al., 2012) throughout April 2017.

### Identification of Variants With High Allele Frequencies in Any Ancestry

We compared the allele frequency of each variant among different ancestries by searching gnomAD version 2.0, TWB, and iJGVD. gnomAD was first released as ExAC in 2013 (Lek et al., 2016), and was expanded to 123,136 exomes and 15,496 genomes in 2017. TWB was released in 2014, and is composed of whole-genome sequencing data from 997 unrelated Han Chinese (HC), and genome-wide association study data from 16,036 unrelated HC (Fan et al., 2008). No subjects with BrS were enrolled in the TWB. iJGVD was first released in 2014 (Nagasaki et al., 2015), and consists of whole-genome sequencing data from 3,554 Japanese subjects (3KJPNv2) obtained in June 2018.

According to the clinical genetic interpretation method proposed by Whiffin et al. (2017), in addition to considering the disease prevalence and penetrance, it is also necessary to consider the allelic contribution made by each pathogenic variant. Accordingly, the maximal credible allele frequency of a pathogenic variant is as follows:

Maximal credible allele frequency = (prevalence×maximal allelic contribution)/penetrance

Brugada syndrome has the highest prevalence (∼0.12%) in East Asian populations (Mizusawa and Wilde, 2012; Juang et al., 2015), with a genetic yield rate of approximately 30–35% (Juang and Horie, 2016), and the contribution of a single variant is no more than 1% (Kapplinger et al., 2010). Moreover, BrS-associated variants may have a penetrance of approximately 16–32.7% according to familial co-segregation analysis (Priori et al., 2000; Hong et al., 2004). Therefore, the allele frequency of a pathogenic variant of BrS is expected to be lower than 0.000075, implying that the pathogenicity of any variants with allele frequencies higher than this value may require careful interpretation. However, *in vitro* studies have revealed that some variants are associated with functional alterations (Matsusue et al., 2016). Such cases should be treated as disease predisposition alleles rather than totally excluding their pathogenic role. Thus, we used a conservative cut-off of 0.001, at least 10 times higher than the frequency mentioned above, which may have prevented the exclusion of some true pathogenic variants from the subsequent phenotype-driven analysis due to too strict a cut-off. The cut-off set by us in the present study was not very strict, and was reasonable as the first step for the reclassification of previously reported pathogenic variants of BrS across different populations.

### Study Cohort

The present study complied with governmental laws and regulations and was carried out in accordance with the Good Practice guidelines provided by Research Ethics Committee B of the National Taiwan University Hospital. All subjects provided written informed consent in accordance with the Declaration of Helsinki. The protocol was approved by Research Ethics Committee B of the National Taiwan University Hospital. We enrolled 124 consecutive unrelated BrS patients from the Han Chinese population of Taiwan between 1998 and 2017 (SADS-TW BrS registry) (Wu et al., 2018). The majority (89.4%) of the patients were male, and their mean age was 44.7 years old. The diagnosis of BrS was in accordance with the expert consensus of the Heart Rhythm Society (HRS), the European Heart Rhythm Association (EHRA), and the Asia-Pacific Heart Rhythm Society (APHRS) (Antzelevitch et al., 2005a; Priori et al., 2013). The Brugada electrocardiographic (ECG) patterns are composed of three types as follows: type 1 ECG has coved-type ST-segment elevation with J point elevation >0.2 mV, followed by a negative T wave; type 2 ECG has saddle-back ST-segment elevation with J point elevation >0.2 mV, followed by a gradually descending ST-segment elevation >0.1 mV and a positive or biphasic T wave; and type 3 ECG has either a saddle-back or coved appearance but with ST-segment elevation <0.1 mV. Only spontaneous or drug-induced type 1 ECG is diagnostic, while type 2 or 3 ECG is suspicious and should undergo drug provocation test by sodium channel blocker. The demographic data were recorded, including gender, age, initial presentation, family history of sudden cardiac death or BrS and electrocardiographic parameters.

### Sanger’s Sequencing

With permission from patients, 10 ml of peripheral venous blood was used for genetic analysis. We used deoxyribonucleic acid (DNA) extraction kit (Qiagen company) to extract DNA from the buffy coat of patients’ peripheral blood. *SCN5A* variants were screened by Sanger’s sequencing. We investigated the prevalence and clinical characteristics of the patients with these reclassified variants in our 124 consecutive unrelated BrS cohort.

### Statistical Analysis

Chi-square or Fisher’s exact tests were used to compare categorical variables. A two-tailed *p*-value < 0.05 was considered statistically significant. Statistical analysis was performed using IBM SPSS Statistics for Windows (version 22.0) (Armonk, NY, United States).

## Results

**FIGURE 1:**
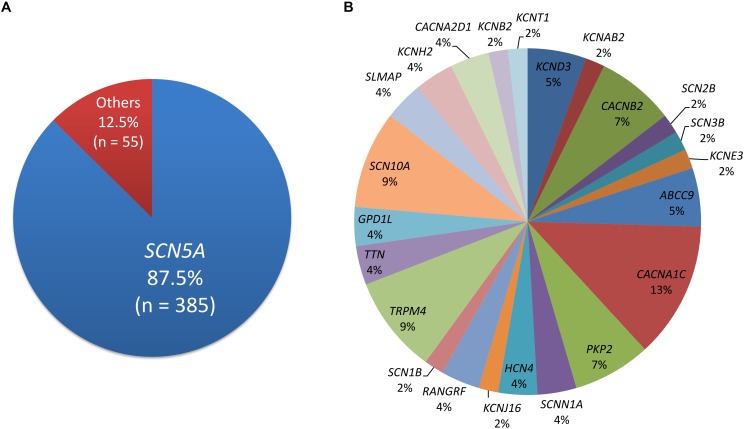
The distribution of pathogenic and likely pathogenic variants. **(A)** *SCN5A* accounts for 87.5%. **(B)** The remaining genes.

Four-hundred and five disease-causing mutations of BrS were reported in HGMD, and 45 pathogenic and 23 likely pathogenic variants were reported in ClinVar. There were 440 variants in total, with 33 overlapping variants. A total of 385 (87.5%) variants were in the SCN5A gene, followed by the CACNA1C, SCN10A, and TRPM4 genes (Figure 1). Twenty-three (6.0%) SCN5A variants were in the voltage-sensing domain (VSD), 52 (13.5%) were in the pore-forming domain, 53 (13.8%) were in other transmembrane domains, 114 (29.6%) were in the extracellular domain, and 143 (37.1%) were in the cytoplasmic domain.

**FIGURE 2:**
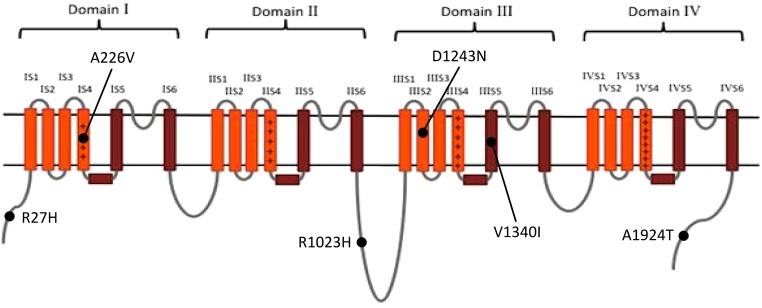
The distribution of the previously reported *SCN5A* variants with allele frequency ≥0.001 in at least one ancestry. Brown: pore-forming domain; +: voltage-sensing domain. Data from gnomAD, iJGVD, and Taiwan Biobank.

Of the 440 previously reported BrS mutations, 18 (4.1%) variants had an allele frequency ≥0.001 in at least one ancestry (Table 1): 17 from HGMD and 1 from ClinVar. There was no significant difference in the percentages of variants found in these two databases (HGMD 4.2% versus ClinVar 1.5%, *p* = 0.4915). All of the original studies used internal controls with 50–700 healthy subjects; four of them used two controls (2: internal controls and ESP6500; 2: internal controls and 1000G). None of the original studies used greater than 3 controls. Eleven of the 18 variants (61.1%) in the previous 13 studies used the Caucasian population as healthy controls. These variants included 6 SCN5A and 12 non-SCN5A variants. There were more previously reported non-SCN5A variants with allele frequencies ≥0.001 than SCN5A variants (21.8 versus 1.6%, *p* < 0.0001). Among the SCN5A variants with allele frequencies ≥0.001, one was located in the VSD, one in the pore-forming domain, one in another transmembrane domain, and the other three variants were in the cytoplasmic domain (Figure 2). The variants with allele frequencies <0.001 across all ancestries are listed in Supplementary Table 1.

**Table 1:** The 18 of the 440 reported pathogenic variants in Brugada syndrome showed allele frequencies ≥0.001 in at least one ancestry.

Gene	Transcript	ΔNA	ΔAA	dbSNP	Controls of the original study	HGMD	gnomAD (total *n* = 138.632)							3KJPNv2 (*n* = 3,554)	TWB (*n* = 997)	SIFT	Polyphen-2
							NFE (*n* = 63,369)	FIN (*n* = 12,897)	AMR (*n* = 17,210)	AFR (*n* = 12,020)	JEW (*n* = 5,076)	EAS (*n* = 9,435)	SAS (*n* = 15,391)				
**Variants from HGMD**																	
ABCC9	NM_005691.3	3589C > T	R1197C	rs778849288	>200 Caucasians	DM (Hu et al., 2014b)	0.000024	0	0	0	0	0.001431^∗^	0.000032	0	0.002008^∗^	Damaging	Probably damaging
CACNA1C	NM_000719.6	6388G > A	D2130N	rs199473392	>200 Caucasians	DM (Burashnikov et al., 2010)	0.000046	0	0	0	0.002504^∗^	0	0	0	0	Damaging	Possibly damaging
HCN4	NM_005477.2	2522C > T	S841L	rs200546024	>700 Caucasians, 1000G	DM (Crotti et al., 2012)	0.000058	0	0.000058	0.000085	0.001802^∗^	0	0	0	0	Damaging	Probably damaging
KCNB2	NM_004770.2	1564G > A	E522K	rs745516217	500 Han Chinese	DM (Juang et al., 2014)	0.000024	0	0	0	0	0.001538^∗^	0	0.0013^∗^	0.001003^∗^	Tolerated	Probably damaging
KCNT1	NM_020822.2	3317G > A	R1106Q	rs561255614	500 Han Chinese	DM (Juang et al., 2014)	0.000062	0	0	0.000053	0	0.004356^∗^	0.000327	0.0027^∗^	0.004692^∗^	Damaging	Probably damaging
PKP2	NM_004572.3	1093A > G	M365V	rs143900944	200 Caucasians, ESP	DM (Cerrone et al., 2014)	0.000237	0	0	0	0	0	0.001137^∗^	0	0	Tolerated	Benign
RANGRF	NM_016492.4	181G > T	E61^∗^	rs140704891	300 Spanish	DM (Selga et al., 2015)	0.005406^∗^	0.01116^∗^	0.001018^∗^	0.000587	0.000297	0	0.001657^∗^	0	0	NA	NA
SCN3B	NM_018400.3	328G > A	V110I	rs147205617	480 Japanese	DM (Ishikawa et al., 2013)	0.000032	0	0.000029	0.000583	0	0.002438^∗^	0.000455	0.0025^∗^	0.003009^∗^	Tolerated	Probably damaging
SCN5A	NM_198056.2	80G > A	R27H	rs199473045	400 Caucasians	DM (Priori et al., 2002)	0.000024	0	0.001569^∗^	0.000208	0	0.000159	0	0	0	Damaging	Probably damaging
SCN5A	NM_198056.2	677C > T	A226V	rs199473561	400 Caucasians	DM (Priori et al., 2002)	0.000008	0	0	0	0	0.001357^∗^	0	0	0.001508^∗^	Damaging	Probably damaging
SCN5A	NM_198056.2	3068G > A	R1023H	rs199473592	200 Japanese; ESP	DM (Watanabe et al., 2013)	0.000048	0	0.000174	0.000042	0	0.000053	0.001887^∗^	0	0	Tolerated	Benign
SCN5A	NM_198056.2	3727G > A	D1243N	rs199473599	649 white, 651 non-white subjects, 200–400 Caucasians	DM (Kapplinger et al., 2010)	0.000024	0.000078	0.000116	0	0.002758^∗^	0.000053	0.000065	0.0001	0	Damaging	Probably damaging
SCN5A	NM_198056.2	4018G > A	V1340I	rs199473605	649 white, 651 non-white subjects (blacks, Asians, Hispanics, and others), 200–400 Indians	DM (Kapplinger et al., 2010)	0.000024	0	0	0.000042	0	0.000371	0.000065	0	0.001003^∗^	Damaging	Probably damaging
SCN10A	NM_006514.3	41G > T	R14L	rs141207048	>200 Caucasians	DM (Hu et al., 2014a)	0.002776^∗^	0.000931	0.000872	0.000375	0.009374^∗^	0	0.000162	0	0	Damaging	Possibly damaging
SCN10A	NM_006514.3	3803G > A	R1268Q	rs138832868	>200 Caucasians	DM (Hu et al., 2014a)	0.003162^∗^	0.002957^∗^	0.000436	0.000542	0.000592	0.000319	0.000033	0.0001	0.000502	Damaging	Probably damaging
SCNN1A	NM_001038.5	1049G > A	R350Q	rs534158738	500 Han Chinese	DM (Juang et al., 2014)	0.000036	0.000066	0	0	0.000465	0.000033	0	0.001	0.001505^∗^	Damaging	Probably damaging
SLMAP	NM_007159.3	2129A > C	E710A	rs765326482	94–380 Japanese, 1000G	DM (Ishikawa et al., 2012)	0	0	0	0	0	0.000988	0	0.0014^∗^	0	Tolerated	Possibly damaging
**Variants from ClinVar**																	
Gene	Transcript	ΔNA	ΔAA	dbSNP	Ethnicity of the original study	ClinVar (reported date)	gnomAD (total *n* = 138.632)							3KJPNv2 (*n* = 3,554)	TWB (*n* = 997)	SIFT	Polyphen-2
							NFE (*n* = 63,369)	FIN (*n* = 12,897)	AMR (*n* = 17,210)	AFR (*n* = 12,020)	JEW (*n* = 5,076)	EAS (*n* = 9,435)	SAS (*n* = 15,391)				
SCN5A	NM_198056.2	5770G > A	A1924T	rs137854603	50 Caucasians	P/LP (2016/07/07) (Rook et al., 1999)	0.000016	0	0.000087	0	0.001084^∗^	0	0	0	0	Tolerated	Possibly damaging


*1000G, 1000 genomes project; AFR, African; AMR, Latino; ΔAA, amino acid change; ΔNA, nucleotide change; DM, disease-causing mutation; EAS, East Asian; ESP, NHLBI Grand Opportunity Exome Sequencing Project; NFE, non-Finnish European; FIN, Finnish; HGMD, The Human Gene Mutation Database; JEW, Ashkenazi Jewish; 3KJPNv2, Japan Biobank; LP, likely pathogenic; NA, not available; P, pathogenic; SAS, South Asian; TWB, Taiwan Biobank; ^∗^≥0.001.*

With regard to ancestral differences, 14 (77.8%) variants had allele frequencies ≥0.001 only in Asian population, including all the non-SCN5A variants except one RANGRF variant and two SCN10A variants. The allele frequencies of RANGRF p.E61^∗^ were ≥0.001 across all ancestries except for African, whereas the allele frequencies of SCN5A p.R27H were ≥0.001 only in American ancestries. These findings demonstrate that ancestral differences impact genetic predisposition in BrS.

Five variants had allele frequencies ≥0.001 in 1000G or ESP, but they were lower in gnomAD (Supplementary Table 2). Because a larger database is more representative of the population frequency, the results from gnomAD should be more reliable than those reported previously.

Regarding the types of mutations, there were 40 nonsense (9.1%), 73 frameshift (16.6%), 23 splice site (9.1%), and 304 missense (69.1%) mutations among the previously reported 440 BrS mutations. The percentages of the 18 variants with minor allele frequency >0.001 in at least one ancestry within each type of mutation were: 0% (0/73) in the frameshift mutations, 2.5% (1/40) in the nonsense mutations, 0% (0/23) in the splice site mutations, and 5.6% (17/304) in the missense mutations. There was a significantly lower percentage of radical mutations (frameshift plus nonsense) than missense mutations (0.9 versus 5.6%, *p* = 0.032).

In our SADS-TW BrS registry cohort, for the variants with minor allele frequencies ≥0.001, two SCN5A variants (p.A226V and p.V1340I) (0.45%) were identified in 5 patients (4%) (Table 1). Of those patients, 4 carried p.A226V and one had p.V1340I. The clinical characteristics of the patients are shown in Table 2. Among the three patients carrying SCN5A p.A226V underwent the electrophysiological examinations, ventricular tachycardia/fibrillation was induced in two patients. In the other hand, we also found 10 pathogenic or likely pathogenic *SCN5A* variants with minor allele frequencies <0.001 in 10 patients with BrS (8.1%), for which the pathogenicity was determined according to the American College of Medical Genetics and Genomics (ACMG) criteria (Richards et al., 2015).

**Table 2:** Clinical characteristics of the patients (SADS-TW BrS registry) carrying previously reported pathogenic variants in HGMD with allele frequencies ≥0.001 in at least one ancestry.

No.	Presentation	Gender	Age at first^3^	Occurrence of type 1 BrP	Family history of SCD	Electrophysiology study	Amino acid change	dbSNP	HGMD	gnomAD_EAS	TWB	Reference
1	Syncope	Male	23	Drug-induced	No	Inducible Vf	A226V	rs199473561	DM	0.001357	0.001508	Priori et al., 2002; Tan et al., 2016
2	Asymptomatic	Male	54	Drug-induced	Yes	Inducible Vf						
3	Chest pain	Male	74	Spontaneous	No	N/A						
4	Syncope	Male	30	Drug-induced	No	No inducible Vf						

5	Syncope	Male	51	Spontaneous	No	N/A	V1340I	rs199473605	DM	0.000371	0.001003	Samani et al., 2009; Kapplinger et al., 2010


*BrP, Brugada-type electrocardiographic pattern; BrS, Brugada syndrome; DM, disease-causing mutation; EAS, East Asian; N/A, not available; SCD, sudden cardiac death; TWB, Taiwan Biobank; Vf, ventricular fibrillation. Transcript: NM_198056.2.*

## Discussion

In dealing with a heritable disease, the identification of the causative genetic variant is important for familial consultation, because it can aid risk stratification in a family and eliminate the anxiety of unaffected members. In the era of precision medicine, misinterpretation of variants may lead to incorrect genetic diagnoses and ineffective treatment strategies.

Brugada syndrome is characterized by the loss of the epicardial action potential dome in the right ventricular outflow tract (Antzelevitch, 2001); thus, variants that alter channel function are potential pathogenic variants. Given that BrS is a rare disease, the allele frequency of each pathogenic variant would be expected to be lower than its prevalence. That is, a variant with a functional alteration but a relatively high allele frequency may not be pathogenic by itself, or may only increase susceptibility to the disease.

**FIGURE 3:**
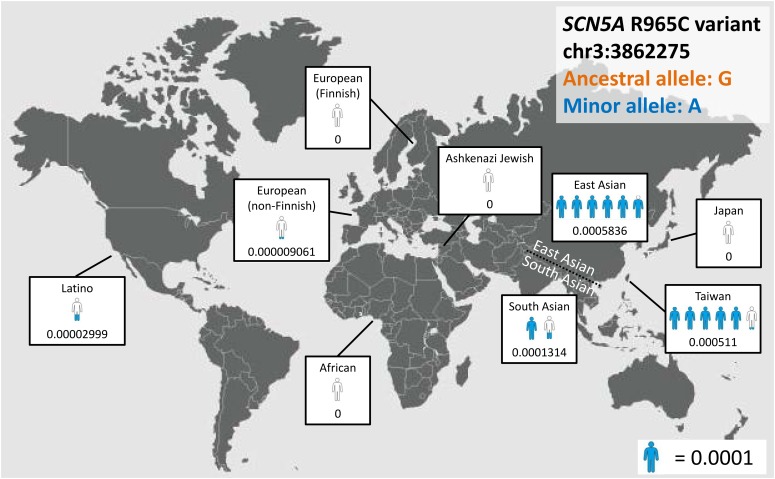
*SCN5A*, c.2893C > T, p.R965C as an example of ancestral differences in allele frequency.

With the development of next-generation sequencing (NGS), population frequency studies can be conducted faster and on a larger scale, and the pathogenicity of variants can be evaluated on the basis of ancestry-specific references. For example, the *SCN5A* p.R1193Q substitution, which accelerates the inactivation of sodium channels (Vatta et al., 2002), is now considered to be a disease predisposition allele rather than a causative mutation in Asian populations; its allele frequency in this ancestry is as high as 0.05 (Matsusue et al., 2016), which is much higher than the disease prevalence rate. Another example involves *SCN5A* c.2893C > T (p.R965C), which exhibits major differences in allele frequency among ancestries (Figure 3). These findings support the importance of establishing a large, population-specific database of allele frequency, which could be the best reference for determining the rarity of variants.

In the present study, we identified 18 variants with minor allele frequencies ≥0.001 in at least one ancestry, indicating that they may be common variants in these populations. In the poly-ancestry population databases, the number of participants in gnomAD is larger than that in 1000G (poly-ancestry) and ESP6500 (European ancestry and African Americans only) whereas in the population-specific databases, TWB (Han Chinese) and iJGVD (Japanese) are probably larger than most internal controls from an individual institution. As a result, the allele frequency in gnomAD, TWB and iJGVD were more approximate to the true allele frequencies of each ancestry. Importantly, 14 (77.8%) of the variants had allele frequencies ≥0.001 only in Asian population. This may imply the distinct genetic background of Asian BrS patients and a different pathogenicity of the same variants in BrS patients across populations. Some variants with definite effects on transmembrane currents, or those predicted to be deleterious by *in silico* analysis, may be reclassified as disease predisposition alleles; however, the pathogenicity of other variants should be subjected to further careful investigation. Since most of these studies used Caucasian population as healthy controls, the MAF in other ancestries could not be known. Therefore, a large database collected from all ancestries is crucial, and variants with allele frequencies <0.001 across all ancestries (European, African, American, and Asian) are much more likely to be pathogenic.

Our study revealed that significantly more previously reported non-*SCN5A* variants than *SCN5A* variants should be reclassified. This finding is consistent with the findings of Le Scouarnec et al. (2015), who showed that there was a higher percentage of BrS cases with rare functional variants in *SCN5A* than in non-*SCN5A* genes, compared with internal controls. There are several possible explanations. First, there have been many comprehensive *in vitro* electrophysiological functional studies of *SCN5A*. The authors of all such studies investigated each variant repeatedly and rigorously, and were therefore able to exclude any variant for which pathogenicity could not be demonstrated. However, studies regarding other genes have usually been small-scale and of limited scope, and may not have excluded some benign variants. Second, *SCN5A* plays an important role in cardiac development and function; thus, non-synonymous variants that alter protein function may directly enhance disease development. Thus, non-*SCN5A* variants should be interpreted carefully.

The substitution of the amino acids that constitute the pore-forming domain of SCN5A may alter its gating function; therefore, this domain should be more highly conserved in evolution across different ancestries than other regions. Alterations to the pore function may be less well tolerated than changes in other regions, which could explain the consistently low allele frequencies of pore-forming domain variants among the databases examined.

Regarding the types of mutations, only 0.9% of the nonsense and frameshift mutations had allele frequencies ≥0.001 in at least one ancestry (European, African, American, or Asian), a significantly lower percentage than that of the missense mutations (5.6%). This difference may imply that BrS-associated nonsense and frameshift mutations are much more likely to be pathogenic, which may be attributed to critical alterations in protein function in these types of mutations.

We found that 4% of the patients with BrS in our cohort carried *SCN5A* variants with minor allele frequencies ≥0.001. Of these patients, four had p.A226V and one had p.V1340I. *SCN5A* p.A226V is located in the VSD, and p.V1340I is located in the pore-forming domain; both reduce the sodium current in patch-clamp studies (Samani et al., 2009; Tan et al., 2016). However, in a Singaporean family reported by Tan et al., the father carrying p.A226V did not show type 1 Brugada ECG after flecainide infusion. This suggested that the pathogenicity of p.A226V may be questionable in the East Asian population, demonstrating that considering ancestral difference and clinical data is as important as functional studies when we interpret the pathogenicity of a genetic variant.

There are several possible reasons why a variant that causes functional alterations *in vitro* could be a disease predisposition allele in at least some ancestries. First, because protein–protein interactions are complicated in living cells, the effects of variants in transfected cells may not always persist *in vivo*, where a gene that does not encode an ion channel may nevertheless alter the current by interacting with that channel (Cerrone et al., 2014). Second, a pathogenic variant may be masked by another variant (Marangoni et al., 2011), and an ancestry with an abundance of such protective variants could be resistant to this pathogenic variant. Third, deoxyribonucleic acid (DNA) methylation is known to regulate gene expression and is highly divergent among ancestries (Fraser et al., 2012). Therefore, functional studies are essential, and ancestral differences should be taken into account when judging the pathogenicity of a novel variant.

### Limitations

The present study has limitations. First, more evidences emerged now that the inheritance underlying BrS may be complex with multiple genes and possibly some environmental factors contributing to the phenotype, and some common variants may also be contributive (Bezzina et al., 2013; Abriel, 2015; Behr et al., 2015). Therefore, we may not be able to exclude possible contribution to pathogenicity of a variant in polygenic model solely because of its high allele frequency. Second, because electrocardiograms are generally not available in the public control databases, it is possible that some asymptomatic individuals carrying pathogenic BrS-associated variants might be enrolled as “apparently healthy” controls in the public databases. However, considering Brugada syndrome is a rare disease with the prevalence of 12/10,000 (Southeast Asia populations) and 5/10,000 (Caucasians) in the general populations, and healthy controls are usually enrolled randomly from a general population, the expected contribution from the minor allele of BrS-associated variants to the public control databases could be very low (0–0.06%). Third, although TWB and iJGVD are the population-specific references for Han Chinese and Japanese populations, respectively, the number of the samples in the two references are relatively small compared with gnomAD. Therefore, the representativity for the two populations and the power of excluding benign variants may also be a concern. Fourth, if a potentially pathogenic BrS-related variant is “protected” by other genes or by epigenetic effects, its pathogenic phenotype may be attenuated. As a result, its allele frequency in a population may be relatively high. However, this variant may remain pathogenic in other populations or segments of the population; for example, for one gender or in certain ancestries. In such a case, the variant would have been considered to have “questionable pathogenicity” in our study and would require periodic re-evaluation.

In conclusion, the allele frequencies of BrS variants differ significantly with ancestry. This finding should be taken into consideration before judging a variant as pathogenic, particularly in the case of non-*SCN5A* variants, which may be pathogenic in some ancestries but only disease-predisposing in others. Besides, in-house controls and small-scale databases of population frequency have less power to exclude benign variants; large-scale super-controls, such as gnomAD and ancestry-specific databases, could be used to address this issue.

## Author Contributions

J-MJ and CA contributed to the conception and design of the study. Y-BL, L-TH, and H-CH collected the data. T-PL and L-PL contributed to the data analysis. C-YC, S-FY, and C-KW wrote the manuscript. L-YL and J-JH supervised the whole project. All authors read and approved the final manuscript.

## Conflict of Interest Statement

The authors declare that the research was conducted in the absence of any commercial or financial relationships that could be construed as a potential conflict of interest.

## Footnotes

1 https://www.ncbi.nlm.nih.gov/clinvar/

2 http://www.hgmd.cf.ac.uk/ac/index.php
