# Supplementary material for: Impact of Ancestral Differences and Reassessment of the Classification of Previously Reported Pathogenic Variants in Patients With Brugada Syndrome in the Genomic Era: A SADS-TW BrS Registry
Source: Front Genet. 2019 Jan 4;9:680. doi: 10.3389/fgene.2018.00680 (PMC6328444; doi:10.3389/fgene.2018.00680)
Supplement: Supplementary file 1 [file Table_1.doc]

**Supplementary Note**

**Impact of ancestral differences and reassessment of the classification of previously reported pathogenic variants for patients with Brugada syndrome in the genomic era: A SADS-TW BrS Registry**

**Short title:** Brugada syndrome in different ancestries

Ching-Yu Julius Chen^1^, Tzu-Pin Lu^2^, Lian-Yu Lin^1^, Yen-Bin Liu^1^, Li-Ting Ho^1^, Hui-Chun Huang^1^, Ling-Ping Lai, MD^1^, Juey-Jen Hwang^1^, Shih-Fan Sherri Yeh^3^, Cho-Kai Wu^1^, Jyh-Ming Jimmy Juang^1*^, Charles Antzelevitch^4^

^1^Cardiovascular Center and Division of Cardiology, Department of Internal Medicine, National Taiwan University Hospital, Taipei, Taiwan

^2^Institute of Epidemiology and Preventive Medicine, Department of Public Health, National Taiwan University, Taipei, Taiwan

^3^Department of Environmental and Occupational Medicine, National Taiwan University Hospital, Taipei, Taiwan

^4^Lankenau Institute for Medical Research, Wynnewood, Pennsylvania, Lankenau Heart Institute, Wynnewood, PA and Sidney Kimmel Medical College of Thomas Jefferson University, Philadelphia, PA USA

To whom correspondence should be addressed: *Jyh-Ming Jimmy Juang, MD, PhD

Cardiovascular Center and Division of Cardiology, Department of Internal Medicine, National Taiwan University Hospital and College of Medicine, National Taiwan University, Taipei, 10002, Taiwan

No.7, Chung Shan S. Rd, Zhong-Zheng Dist., Taipei, Taiwan

Telephone: +886-972651396

E-mail: jjmjuang@ntu.edu.tw

Supplementary Table 1: The variants with allele frequency <0.001 across the ancestries.

| Gene | Transcript | ΔNA | ΔAA | gnomAD | | | | | | | 3KJPNv2 | TWB |
| --- | --- | --- | --- | --- | --- | --- | --- | --- | --- | --- | --- | --- |
|  |  |  |  | NFE | FIN | AMR | AFR | JEW | EAS | SAS |  |  |
| ABCC9 | NM_005691.3 | c.2197A>G | p.N733D | 3.58E-05 | 0 | 0 | 0 | 0 | 0 | 0 | 0 | 0 |
| ABCC9 | NM_005691.3 | c.4205C>G | p.S1402C | 3.96E-05 | 0 | 0 | 0 | 0 | 0 | 0 | 0 | 0 |
| CACNA1C | NM_000719.6 | c.116C>T | p.A39V | 0 | 0 | 0 | 0 | 0 | 0 | 0 | 0 | 0 |
| CACNA1C | NM_000719.6 | c.898A>G | p.N300D | 4.5E-05 | 0 | 5.98E-05 | 0 | 0 | 0 | 0 | 0 | 0 |
| CACNA1C | NM_000719.6 | c.1640A>G | p.N547S | 0 | 0 | 0 | 0 | 0 | 0 | 0 | 0 | 0 |
| CACNA1C | NM_000719.6 | c.3343G>A | p.E1115K | 0 | 0 | 0 | 0 | 0 | 0 | 0 | 0 | 0 |
| CACNA1C | NM_000719.6 | c.5339G>A | p.R1780H | 9.32E-06 | 0 | 3.01E-05 | 0 | 0 | 5.86E-05 | 0 | 0 | 0 |
| CACNA1C | NM_000719.6 | c.5487_5501dup15 | GACGTCT^1833CAGgaggagacgtctcagGATGAGACCT | 0 | 0 | 0 | 0 | 0 | 0 | 0 | 0 | 0 |
| CACNA2D1 | NM_000722.3 | c.1648G>T | p.D550Y | 1.74E-04 | 0 | 0 | 4.2E-05 | 0 | 0 | 0 | 0 | 0 |
| CACNA2D1 | NM_000722.3 | c.2867C>A | p.S956Y | 2.7E-05 | 0 | 0 | 0 | 0 | 0 | 0 | 0 | 0 |
| CACNB2 | NM_201590.2 | c.32C>T | p.T11I | 0 | 0 | 0 | 0 | 0 | 0 | 0 | 0 | 0 |
| CACNB2 | NM_201590.2 | c.1018G>A | p.V340I | 1.08E-04 | 0 | 0 | 0 | 0 | 0 | 0 | 0 | 0 |
| CACNB2 | NM_201590.2 | c.1442C>T | p.S481L | 0 | 0 | 0 | 0 | 0 | 0 | 0 | 0 | 0 |
| CACNB2 | NM_201590.2 | c.1497G>C | p.E499D | 1.6E-05 | 0 | 1.74E-04 | 0 | 0 | 0 | 0 | 0 | 0 |
| HCN4 | NM_005477.2 | c.1209_1209+1insGTGA | NA | 0 | 0 | 0 | 0 | 0 | 0 | 0 | 0 | 0 |
| KCNAB2 | NM_003636.3 | c.35G>A | p.R12Q | 0 | 0 | 3.0E-05 | 0 | 0 | 1.16E-04 | 3.2E-05 | 0 | 0 |
| KCND3 | NM_004980.4 | c.1174G>A | p.V392I | 0 | 0 | 0 | 0 | 0 | 0 | 0 | 0 | 0 |
| KCND3 | NM_004980.4 | c.1348C>T | p.L450F | 1.28E-04 | 0 | 5.9E-05 | 0 | 6.97E-04 | 0 | 1.35E-04 | 0 | 0 |
| KCND3 | NM_004980.4 | c.1798G>A | p.G600R | 1.1E-04 | 0 | 8.72E-06 | 0 | 0 | 0 | 0 | 0 | 0 |
| KCNE3 | NM_005472.4 | c.296G>A | p.R99H | 9.5E-05 | 7.8E-05 | 2.9E-05 | 8.3E-05 | 0 | 1.59E-04 | 1.95E-04 | 0 | 0 |
| KCNH2 | NM_000238.3 | c.2350C>T | p.R784W | 2.38E-05 | 0 | 0 | 0 | 0 | 0 | 0 | 0 | 0 |
| KCNH2 | NM_000238.3 | c.455C>T | p.T152I | 0 | 0 | 0 | 0 | 0 | 0 | 0 | 0 | 0 |
| KCNJ16 | NM_018658.2 | c.886A>G | p.S296G | 0 | 0 | 0 | 0 | 0 | 0 | 0 | 0 | 0 |
| PKP2 | NM_004572.3 | c.548G>A | p.S183N | 5.5E-05 | 4.0E-05 | 3.0E-05 | 0 | 9.86E-04 | 1.06E-04 | 0 | 7.0E-04 | 0 |
| PKP2 | NM_004572.3 | c.1576A>G | p.T526A | 2.53E-04 | 0 | 2.9E-05 | 0 | 0 | 0 | 3.2E-05 | 0 | 0 |
| PKP2 | NM_004572.3 | c.1904G>A | p.R635Q | 8.96E-06 | 0 | 0 | 0 | 0 | 0 | 3.25E-05 | 0 | 0 |
| RANGRF | NM_016492.4 | c.249G>C | p.E83D | 3.95E-05 | 0 | 8.72E-05 | 0 | 0 | 0 | 0 | 0 | 0 |
| SCN10A | NM_006514.3 | c.599C>T | p.A200V | 1.26E-04 | 0 | 1.45E-04 | 8.3E-05 | 9.9E-05 | 0 | 1.3E-04 | 0 | 0 |
| SCN10A | NM_006514.3 | c.2011A>G | p.I671V | 1.79E-05 | 0 | 0 | 0 | 0 | 0 | 0 | 0 | 0 |
| SCN10A | NM_006514.3 | c.2813_2814delTCinsA | TCCTGC^937CCATtcCCCCAGCCCA | 0 | 0 | 0 | 0 | 0 | 0 | 0 | 0 | 0 |
| SCNN1A | NM_001038.5 | c.1789C>T | p.R597* | 0 | 0 | 0 | 0 | 0 | 4.24E-04 | 3.2E-05 | 0 | 0 |
| SLMAP | NM_007159.3 | c.805G>A | p.V269I | 0 | 0 | 0 | 0 | 0 | 2.04E-04 | 0 | 5.0E-04 | 0 |
| SCN1B | NM_001037.4 | c.448+88G>A | NA | 1.81E-05 | 0 | 0 | 0 | 0 | 0 | 0 | 0 | 0 |
| SCN2B | NM_004588.4 | c.632A>G | p.D211G | 1.79E-05 | 0 | 0 | 0 | 0 | 0 | 0 | 0 | 0 |
| GPD1L | NM_015141.3 | c.247G>A | p.E83K | 3.16E-04 | 0 | 8.7E-05 | 0 | 0 | 0 | 6.5E-05 | 0 | 0 |
| GPD1L | NM_015141.3 | c.839C>T | p.A280V | 1.77E-04 | 0 | 5.8E-05 | 0 | 0 | 0 | 1.63E-04 | 0 | 0 |
| SCN5A | NM_198056.2 | c.1595T>G | p.F532C | 0 | 0 | 0 | 0 | 0 | 2.33E-04 | 0 | 5.0E-04 | 0 |
| SCN5A | NM_198056.2 | c.2893C>T | p.R965C | 1.0E-05 | 0 | 3.0E-05 | 0 | 0 | 5.84E-04 | 1.31E-04 | 0 | 0 |
| SCN5A | NM_198056.2 | c.3157G>A | p.E1053K | 1.27E-04 | 0 | 2.62E-04 | 4.2E-05 | 0 | 0 | 0 | 0 | 0 |
| SCN5A | NM_198056.2 | c.3622G>A | p.E1208K | 2.7E-05 | 0 | 0 | 0 | 0 | 0 | 0 | 2.0E-04 | 0 |
| SCN5A | NM_198056.2 | c.3784G>A | p.G1262S | 1.6E-05 | 0 | 2.9E-05 | 0 | 0 | 5.3E-05 | 1.3E-04 | 0 | 0 |
| SCN5A | NM_198056.2 | c.4859C>T | p.T1620M | 0 | 0 | 0 | 0 | 0 | 0 | 0 | 3.0E-04 | 0 |
| SCN5A | NM_198056.2 | c.6010_6012dupTTC | CGCCGAC^2004TTCttcCCCCCTTCTC | 0 | 0 | 0 | 0 | 0 | 0 | 0 | 0 | 0 |
| SCN5A | NM_198056.2 | A4580G G4705C | p.K1527R, p.A1569P | 0 | 0 | 0 | 0 | 0 | 0 | 0 | 0 | 0 |
| SCN5A | NM_198056.2 | c.[1535C>T;1673A>G] | p.T512N, p.H558P | 0 | 0 | 0 | 0 | 0 | 0 | 0 | 0 | 0 |
| SCN5A | NM_198056.2 | c.[3694C>T;4859C>T] | p.T1620K | 0 | 0 | 0 | 0 | 0 | 0 | 0 | 0 | 0 |
| SCN5A | NM_198056.2 | c.[694G>A;3922C>T] | p.V232F, p.L1308I | 0 | 0 | 0 | 0 | 0 | 0 | 0 | 0 | 0 |
| SCN5A | NM_198056.2 | c.283G>A | p.V95I | 1.8E-05 | 0 | 0 | 0 | 0 | 1.74E-04 | 9.7E-05 | 0 | 0 |
| SCN5A | NM_198056.2 | c.393-1C>T | NA | 1.34E-04 | 0 | 0 | 0 | 0 | 0 | 0 | 0 | 0 |
| SCN5A | NM_198056.2 | c.436G>A | p.V146M | 2.7E-05 | 0 | 6.3E-04 | 6.6E-05 | 0 | 5.8E-05 | 6.7E-05 | 0 | 0 |
| SCN5A | NM_198056.2 | c.688A>G | p.I230V | 0 | 0 | 0 | 0 | 0 | 0 | 0 | 0 | 0 |
| SCN5A | NM_198056.2 | c.874G>A | p.G292S | 8.0E-06 | 0 | 0 | 4.2E-05 | 0 | 4.24E-04 | 0 | 0 | 0 |
| SCN5A | NM_198056.2 | c.934+4C>T | NA | 4.3E-04 | 3.9E-05 | 0 | 0 | 0 | 0 | 3.3E-05 | 0 | 0 |
| SCN5A | NM_198056.2 | c.955G>A | p.G319S | 8.0E-06 | 0 | 0 | 1.25E-04 | 0 | 0 | 3.25E-04 | 0 | 0 |
| SCN5A | NM_198056.2 | c.959C>A | p.T320N | 0 | 0 | 0 | 0 | 3.05E-04 | 0 | 0 | 0 | 0 |
| SCN5A | NM_198056.2 | c.1007C>T | p.P336L | 0 | 0 | 0 | 0 | 0 | 0 | 0 | 0 | 0 |
| SCN5A | NM_198056.2 | c.1052G>A | p.G351D | 0 | 0 | 0 | 0 | 0 | 0 | 0 | 0 | 0 |
| SCN5A | NM_198056.2 | c.1052G>T | p.G351V | 0 | 0 | 0 | 0 | 0 | 0 | 0 | 0 | 0 |
| SCN5A | NM_198056.2 | c.1058C>T | p.T353I | 0 | 0 | 0 | 0 | 0 | 0 | 0 | 0 | 0 |
| SCN5A | NM_198056.2 | c.1066G>A | p.D356N | 0 | 0 | 0 | 0 | 0 | 0 | 0 | 0 | 0 |
| SCN5A | NM_198056.2 | c.1099C>T | p.R367C | 0 | 0 | 5.81E-05 | 4.17E-05 | 0 | 0 | 0 | 0 | 0 |
| SCN5A | NM_198056.2 | c.1100G>A | p.R367L | 0 | 0 | 0 | 0 | 0 | 0 | 0 | 0 | 0 |
| SCN5A | NM_198056.2 | c.1100G>T | p.R367H | 0 | 0 | 0 | 0 | 0 | 0 | 0 | 0 | 0 |
| SCN5A | NM_198056.2 | c.1106T>A | p.M369K | 0 | 0 | 0 | 0 | 0 | 0 | 0 | 0 | 0 |
| SCN5A | NM_198056.2 | c.1120T>G | p.W374G | 0 | 0 | 0 | 0 | 0 | 0 | 0 | 0 | 0 |
| SCN5A | NM_198056.2 | c.1127G>A | p.R376H | 7.93E-06 | 0 | 2.91E-05 | 4.2E-05 | 0 | 0 | 0 | 0 | 0 |
| SCN5A | NM_198056.2 | c.1156G>A | p.G386R | 0 | 0 | 0 | 0 | 0 | 0 | 0 | 0 | 0 |
| SCN5A | NM_198056.2 | c.1157G>A | p.G386E | 0 | 0 | 0 | 0 | 0 | 0 | 0 | 0 | 0 |
| SCN5A | NM_198056.2 | c.1177_1179delTTC | CATGATC^392TTCttcATGCTTGTCA | 0 | 0 | 0 | 0 | 0 | 0 | 0 | 0 | 0 |
| SCN5A | NM_198056.2 | c.1186G>C | p.V396L | 0 | 0 | 0 | 0 | 0 | 0 | 0 | 0 | 0 |
| SCN5A | NM_198056.2 | c.1187T>C | p.V396A | 0 | 0 | 0 | 0 | 0 | 0 | 0 | 0 | 0 |
| SCN5A | NM_198056.2 | c.1189A>T | p.I397F | 0 | 0 | 0 | 0 | 0 | 0 | 0 | 0 | 0 |
| SCN5A | NM_198056.2 | c.1217A>G | p.N406S | 0 | 0 | 0 | 0 | 0 | 0 | 0 | 0 | 0 |
| SCN5A | NM_000335.4 | c.1231G>A | p.V411M | 0 | 0 | 0 | 0 | 0 | 0 | 0 | 0 | 0 |
| SCN5A | NM_198056.2 | c.1255C>T | p.Q419* | 0 | 0 | 0 | 0 | 0 | 0 | 0 | 0 | 0 |
| SCN5A | NM_198056.2 | c.127C>T | p.R43* | 0 | 0 | 0 | 0 | 0 | 0 | 0 | 0 | 0 |
| SCN5A | NM_198056.2 | c.1315G>A | p.E439K | 0 | 0 | 0 | 0 | 0 | 0 | 0 | 0 | 0 |
| SCN5A | NM_198056.2 | c.1338+2T>A | NA | 0 | 0 | 0 | 0 | 0 | 0 | 0 | 0 | 0 |
| SCN5A | NM_198056.2 | c.1398G>T | p.L466F | 4.49E-05 | 0 | 0 | 0 | 0 | 0 | 0 | 0 | 0 |
| SCN5A | NM_198056.2 | c.1417G>T | p.E473* | 0 | 0 | 0 | 0 | 0 | 0 | 0 | 0 | 0 |
| SCN5A | NM_198056.2 | c.1428_1431delCAAG | AGAGA^475AGAAGcaagAGGAGAAAAC | 0 | 0 | 0 | 0 | 0 | 0 | 0 | 0 | 0 |
| SCN5A | NM_198056.2 | c.1502A>G | p.D501G | 0 | 0 | 0 | 0 | 0 | 0 | 0 | 0 | 0 |
| SCN5A | NM_198056.2 | c.1537delC | CAGCCTC^512ACCcGTGGCCTCAG | 0 | 0 | 0 | 0 | 0 | 0 | 0 | 0 | 0 |
| SCN5A | NM_198056.2 | c.1562delA | ACTTCT^520ATGAaGCCACGTTCC | 0 | 0 | 0 | 0 | 0 | 0 | 0 | 0 | 0 |
| SCN5A | NM_198056.2 | c.1603C>T | p.R535* | 0 | 0 | 0 | 0 | 0 | 5.81E-05 | 0 | 0 | 0 |
| SCN5A | NM_198056.2 | c.1629T>A | p.F543L | 0 | 0 | 0 | 0 | 0 | 0 | 0 | 0 | 0 |
| SCN5A | NM_198056.2 | c.163C>T | p.Q55* | 0 | 0 | 0 | 0 | 0 | 0 | 0 | 0 | 0 |
| SCN5A | NM_198056.2 | c.1651G>A | p.A551T | 0 | 0 | 0 | 0 | 0 | 0 | 0 | 0 | 0 |
| SCN5A | NM_198056.2 | c.1652C>T | p.A551V | 5.53E-05 | 0 | 8.72E-05 | 4.17E-05 | 0 | 5.3E-05 | 0 | 0 | 0 |
| SCN5A | NM_198056.2 | c.1654G>A | p.G552R | 0 | 0 | 0 | 0 | 0 | 0 | 0 | 0 | 0 |
| SCN5A | NM_198056.2 | c.1657G>T | p.E553* | 0 | 0 | 0 | 0 | 0 | 0 | 0 | 0 | 0 |
| SCN5A | NM_198056.2 | c.1663dupG | GGGGAG^554AGCGgAGAGCCACCA | 0 | 0 | 0 | 0 | 0 | 0 | 0 | 0 | 0 |
| SCN5A | NM_198056.2 | c.1700T>A | p.L567Q | 0 | 0 | 0 | 0 | 0 | 0 | 0 | 0 | 0 |
| SCN5A | NM_198056.2 | c.1705C>G | p.R569G | 0 | 0 | 0 | 0 | 0 | 0 | 0 | 0 | 0 |
| SCN5A | NM_198056.2 | c.1705dupC | CCCCTG^568CGCCcGGACCAGTGC | 0 | 0 | 0 | 0 | 0 | 0 | 0 | 0 | 0 |
| SCN5A | NM_198056.2 | c.1717C>T | p.Q573* | 0 | 0 | 0 | 0 | 0 | 0 | 0 | 0 | 0 |
| SCN5A | NM_198056.2 | c.1721delG | AGTGCC^573CAGGgACAGCCCAGT | 0 | 0 | 0 | 0 | 0 | 0 | 0 | 0 | 0 |
| SCN5A | NM_198056.2 | c.1756G>A | p.A586T | 1.8E-05 | 0 | 0 | 6.55E-05 | 0 | 5.8E-05 | 0 | 0 | 0 |
| SCN5A | NM_198056.2 | c.1776C>G | p.N592K | 0 | 0 | 0 | 0 | 0 | 4.64E-04 | 0 | 0 | 0 |
| SCN5A | NM_198056.2 | c.1858C>T | p.R620C | 1.9E-05 | 4.6E-05 | 0 | 0 | 0 | 1.14E-04 | 5.9E-05 | 0 | 0 |
| SCN5A | NM_198056.2 | c.1872dupA | TGTGATG^624CTAaGAGCACCCGC | 0 | 0 | 0 | 0 | 0 | 0 | 0 | 0 | 0 |
| SCN5A | NM_198056.2 | c.1890+5G>A | NA | 0 | 0 | 0 | 0 | 0 | 0 | 0 | 0 | 0 |
| SCN5A | NM_198056.2 | c.1890G>A | p.T632M | 0 | 0 | 4.81E-05 | 0 | 0 | 0 | 0 | 0 | 0 |
| SCN5A | NM_198056.2 | c.1895C>T | p.P640A | 0 | 0 | 0 | 0 | 0 | 0 | 0 | 0 | 0 |
| SCN5A | NM_198056.2 | c.191_193delTGC | TCCAAA^63AAGCtgcCAGATCTCTA | 0 | 0 | 0 | 0 | 0 | 0 | 0 | 0 | 0 |
| SCN5A | NM_198056.2 | c.1918C>G | p.P640A | 0 | 0 | 0 | 0 | 0 | 0 | 3.26E-05 | 0 | 0 |
| SCN5A | NM_198056.2 | c.1936delC | GCTGACC^645TCCcAGGCTCCGTG | 6.68E-05 | 0 | 0 | 0 | 0 | 0 | 0 | 0 | 0 |
| SCN5A | NM_198056.2 | c.1940C>A | p.A647D | 0 | 0 | 0 | 0 | 0 | 0 | 0 | 0 | 0 |
| SCN5A | NM_198056.2 | c.1950_1953delAGAT | CTCCG^649TGTGTagatGGCTTCGAGG | 0 | 0 | 0 | 0 | 0 | 0 | 0 | 0 | 0 |
| SCN5A | NM_198056.2 | c.2024_2025delAG | GTCCCCTCAG_I14E15_ag^676TTAGAGGAGT | 0 | 0 | 0 | 0 | 0 | 0 | 0 | 0 | 0 |
| SCN5A | NM_198056.2 | c.2024-1G>C | NA | 0 | 0 | 0 | 0 | 0 | 0 | 0 | 0 | 0 |
| SCN5A | NM_198056.2 | c.2042A>C | p.H681P | 0 | 0 | 0 | 0 | 0 | 0 | 0 | 0 | 0 |
| SCN5A | NM_198056.2 | c.2047T>G | p.C683G | 0 | 0 | 0 | 0 | 0 | 0 | 0 | 0 | 0 |
| SCN5A | NM_198056.2 | c.2092G>T | p.E698* | 0 | 0 | 0 | 0 | 0 | 0 | 0 | 0 | 0 |
| SCN5A | NM_198056.2 | c.210T>G | p.N70K | 6.34E-05 | 0 | 8.95E-05 | 6.55E-05 | 0 | 0 | 0 | 0 | 0 |
| SCN5A | NM_198056.2 | c.2201dupT | CACTC^733TTCATtGGCGCTGGAG | 0 | 0 | 0 | 0 | 0 | 0 | 0 | 0 | 0 |
| SCN5A | NM_198056.2 | c.2182G>A | p.V728I | 9.0E-06 | 0 | 3.0E-05 | 0 | 0 | 0 | 0 | 0 | 0 |
| SCN5A | NM_198056.2 | c.2365G>A | p.V789I | 1.03E-04 | 3.9E-05 | 0 | 4.2E-05 | 0 | 0 | 0 | 0 | 0 |
| SCN5A | NM_198056.2 | c.2441G>A | p.R814Q | 3.2E-05 | 0 | 2.9E-05 | 0 | 0 | 5.3E-05 | 3.3E-05 | 3.0E-04 | 0 |
| SCN5A | NM_198056.2 | c.2553C>A | p.F851L | 0 | 0 | 0 | 0 | 0 | 1.06E-04 | 0 | 0 | 0 |
| SCN5A | NM_198056.2 | c.2893C>T | p.R965C | 1.0E-05 | 0 | 3.0E-05 | 0 | 0 | 5.84E-04 | 1.31E-04 | 0 | 0 |
| SCN5A | NM_198056.2 | c.2989G>A | p.A997T | 1.57E-04 | 0 | 5.9E-05 | 0 | 0 | 0 | 3.3E-05 | 0 | 0 |
| SCN5A | NM_198056.2 | c.2203G>A | p.A735T | 0 | 0 | 0 | 0 | 0 | 0 | 0 | 0 | 0 |
| SCN5A | NM_198056.2 | c.2204C>A | p.A735E | 0 | 0 | 0 | 0 | 0 | 0 | 0 | 0 | 0 |
| SCN5A | NM_000335.4 | c.2204C>T | p.A735V | 0 | 0 | 0 | 0 | 0 | 5.8E-05 | 0 | 0 | 0 |
| SCN5A | NM_198056.2 | c.2236G>A | p.E746K | 1.59E-05 | 0 | 5.82E-05 | 8.32E-05 | 0 | 0 | 0 | 0 | 0 |
| SCN5A | NM_198056.2 | c.2254G>A | p.G752R | 0 | 0 | 0 | 0 | 0 | 0 | 3.27E-05 | 0 | 0 |
| SCN5A | NM_198056.2 | c.2273G>A | p.G758E | 0 | 0 | 0 | 0 | 0 | 0 | 0 | 0 | 0 |
| SCN5A | NM_198056.2 | c.2274delG | TCTTC^757ACAGGgATTTTCACAG | 0 | 0 | 0 | 0 | 0 | 0 | 0 | 0 | 0 |
| SCN5A | NM_198056.2 | c.2291T>G | p.M764R | 0 | 0 | 0 | 0 | 0 | 0 | 0 | 0 | 0 |
| SCN5A | NM_198056.2 | c.2317C>T | p.P773S | 0 | 0 | 0 | 0 | 0 | 0 | 0 | 0 | 0 |
| SCN5A | NM_198056.2 | c.2327_2329delACT | CCCCTAC^775TACtacTTCCAACAGG | 0 | 0 | 0 | 0 | 0 | 0 | 0 | 0 | 0 |
| SCN5A | NM_198056.2 | c.2353G>A | p.D785N | 0 | 0 | 0 | 0 | 0 | 0 | 0 | 0 | 0 |
| SCN5A | NM_198056.2 | c.2374dupA | GTCATC^791CTTAaGCCTCATGGA | 0 | 0 | 0 | 0 | 0 | 0 | 0 | 0 | 0 |
| SCN5A | NM_198056.2 | c.2422C>T | p.R808C | 9.01E-06 | 0 | 0 | 0 | 0 | 0 | 3.27E-05 | 0 | 0 |
| SCN5A | NM_198056.2 | c.2432G>A | p.R811H | 0 | 0 | 2.99E-05 | 0 | 0 | 0 | 6.62E-05 | 0 | 0 |
| SCN5A | NM_198056.2 | c.2435_2436+3delTGGTAinsCGCCT | TCCTTC^811CGCCtg_E16I16_gtaCCTGGCTGGA | 0 | 0 | 0 | 0 | 0 | 0 | 0 | 0 | 0 |
| SCN5A | NM_198056.2 | c.2435T>A | p.L812Q | 0 | 0 | 0 | 0 | 0 | 0 | 0 | 0 | 0 |
| SCN5A | NM_198056.2 | c.2465G>A | p.W822* | 0 | 0 | 0 | 0 | 0 | 0 | 0 | 0 | 0 |
| SCN5A | NM_198056.2 | c.2466G>T | p.W822C | 0 | 0 | 0 | 0 | 0 | 0 | 0 | 0 | 0 |
| SCN5A | NM_198056.2 | c.2504C>T | p.S835L | 0 | 0 | 0 | 0 | 0 | 0 | 0 | 0 | 0 |
| SCN5A | NM_198056.2 | c.250G>A | p.D84N | 0 | 0 | 0 | 0 | 0 | 0 | 0 | 0 | 0 |
| SCN5A | NM_198056.2 | c.2516T>C | p.L839P | 0 | 0 | 0 | 0 | 0 | 0 | 0 | 0 | 0 |
| SCN5A | NM_198056.2 | c.2533delG | CCTGACA^844CTGgTGCTAGCCAT | 0 | 0 | 0 | 0 | 0 | 0 | 0 | 0 | 0 |
| SCN5A | NM_198056.2 | c.2541delC | TGGTG^846CTAGCcATCATCGTGT | 0 | 0 | 0 | 0 | 0 | 0 | 0 | 0 | 0 |
| SCN5A | NM_198056.2 | c.2549_2550insTG | CCATC^849ATCGTtgGTTCATCTTT | 0 | 0 | 0 | 0 | 0 | 0 | 0 | 0 | 0 |
| SCN5A | NM_198056.2 | c.2551T>C | p.F851L | 0 | 0 | 0 | 0 | 0 | 0 | 0 | 0 | 0 |
| SCN5A | NM_198056.2 | c.255delC | p.F86Sfs | 0 | 0 | 0 | 0 | 0 | 0 | 0 | 0 | 0 |
| SCN5A | NM_198056.2 | c.2582_2583delTT | ATGCAG^860CTCTttGGCAAGAACT | 0 | 0 | 0 | 0 | 0 | 0 | 0 | 0 | 0 |
| SCN5A | NM_198056.2 | c.2599G>C | p.E867Q | 0 | 0 | 0 | 0 | 0 | 0 | 0 | 0 | 0 |
| SCN5A | NM_198056.2 | c.2599G>T | p.E867* | 0 | 0 | 0 | 0 | 0 | 0 | 0 | 0 | 0 |
| SCN5A | NM_198056.2 | c.2602delC | CTACTCG^867GAGcTGAGGGACAG | 0 | 0 | 0 | 0 | 0 | 0 | 0 | 0 | 0 |
| SCN5A | NM_198056.2 | c.260A>G | p.Y87C | 0 | 0 | 0 | 0 | 0 | 0 | 0 | 0 | 0 |
| SCN5A | NM_198056.2 | c.2613delC | TGAGG^870GACAGcGACTCAGGCC | 0 | 0 | 0 | 0 | 0 | 0 | 0 | 0 | 0 |
| SCN5A | NM_198056.2 | c.2632C>T | p.R878C | 0 | 0 | 0 | 0 | 0 | 0 | 0 | 0 | 0 |
| SCN5A | NM_198056.2 | c.2633G>A | p.R878H | 0 | 0 | 0 | 0 | 0 | 0 | 0 | 0 | 0 |
| SCN5A | NM_198056.2 | c.2657A>C | p.H886P | 6.68E-05 | 0 | 0 | 0 | 0 | 0 | 0 | 0 | 0 |
| SCN5A | NM_198056.2 | c.2669T>C | p.I890T | 0 | 0 | 0 | 0 | 0 | 0 | 0 | 0 | 0 |
| SCN5A | NM_198056.2 | c.2674T>A | p.F892I | 0 | 0 | 0 | 0 | 0 | 0 | 0 | 0 | 0 |
| SCN5A | NM_198056.2 | c.2677C>T | p.R893C | 7.89E-06 | 0 | 0 | 4.16E-05 | 0 | 0 | 0 | 0 | 0 |
| SCN5A | NM_198056.2 | c.2678G>A | p.R893H | 0 | 4.48E-05 | 0 | 0 | 0 | 0 | 0 | 0 | 0 |
| SCN5A | NM_198056.2 | c.2686T>A | p.C896S | 0 | 0 | 0 | 0 | 0 | 0 | 0 | 0 | 0 |
| SCN5A | NM_198056.2 | c.2690G>A | p.G897E | 0 | 0 | 0 | 0 | 0 | 0 | 0 | 0 | 0 |
| SCN5A | NM_198056.2 | c.2701G>A | p.E901K | 0 | 0 | 0 | 0 | 0 | 0 | 0 | 0 | 0 |
| SCN5A | NM_198056.2 | c.2729C>T | p.S910L | 8.96E-06 | 0 | 0 | 0 | 0 | 0 | 0 | 0 | 0 |
| SCN5A | NM_198056.2 | c.2743T>C | p.C915R | 0 | 0 | 0 | 0 | 0 | 0 | 0 | 0 | 0 |
| SCN5A | NM_198056.2 | c.2750T>G | p.L917R | 0 | 0 | 0 | 0 | 0 | 0 | 0 | 0 | 0 |
| SCN5A | NM_198056.2 | c.2780A>G | p.N927S | 0 | 0 | 0 | 0 | 0 | 0 | 0 | 0 | 0 |
| SCN5A | NM_198056.2 | c.2783T>C | p.L928P | 0 | 0 | 0 | 0 | 0 | 0 | 0 | 0 | 0 |
| SCN5A | NM_198056.2 | c.278T>C | p.F93S | 0 | 0 | 0 | 0 | 0 | 0 | 0 | 0 | 0 |
| SCN5A | NM_198056.2 | c.2799_2800delCT | TCCTG^932AATCTctTCCTGGCCTT | 0 | 0 | 0 | 0 | 0 | 0 | 0 | 0 | 0 |
| SCN5A | NM_198056.2 | c.2804T>C | p.L935P | 0 | 0 | 0 | 0 | 0 | 0 | 0 | 0 | 0 |
| SCN5A | NM_198056.2 | c.281T>G | p.I94S | 0 | 0 | 0 | 0 | 0 | 0 | 0 | 0 | 0 |
| SCN5A | NM_198056.2 | c.2850delT | TCACA^949GCCCCtGATGAGGACA | 0 | 0 | 0 | 0 | 0 | 0 | 0 | 0 | 0 |
| SCN5A | NM_198056.2 | c.2894G>A | p.R965H | 0 | 0 | 0 | 0 | 0 | 0 | 0 | 0 | 0 |
| SCN5A | NM_198056.2 | c.2914_2923del10 | GGGCCTG^971CGCtttgtcaagcGGACCACCTG | 0 | 0 | 0 | 0 | 0 | 0 | 0 | 0 | 0 |
| SCN5A | NM_198056.2 | c.2953dupC | TGTGGT^984CTCCcTGCGGCAGCG | 0 | 0 | 0 | 0 | 0 | 0 | 0 | 0 | 0 |
| SCN5A | NM_198056.2 | c.2962C>T | p.R988W | 0 | 0 | 0 | 0 | 0 | 0 | 0 | 0 | 0 |
| SCN5A | NM_198056.2 | c.3005_3012delCCAGCTGC | GGCCAG^1001CTGCccagctgcATTGCCACCC | 0 | 0 | 0 | 0 | 0 | 0 | 0 | 0 | 0 |
| SCN5A | NM_198056.2 | c.3045_3046delGA | CACCC^1014CCAGAgaCGGAGAAGGT | 0 | 0 | 0 | 0 | 0 | 0 | 0 | 0 | 0 |
| SCN5A | NM_198056.2 | c.310C>T | p.R104W | 0 | 4.48E-05 | 0 | 0 | 0 | 0 | 0 | 0 | 0 |
| SCN5A | NM_198056.2 | c.311G>A | p.R104Q | 0 | 0 | 0 | 0 | 0 | 0 | 0 | 0 | 0 |
| SCN5A | NM_000335.4 | c.3142_3154delCCCATCGCTGTGGinsTCTGACTGTGT | p.P1048Sfs | 0 | 0 | 0 | 0 | 0 | 0 | 0 | 0 | 0 |
| SCN5A | NM_198056.2 | c.3157G>A | p.E1053K | 1.27E-04 | 0 | 2.62E-04 | 4.2E-05 | 0 | 0 | 0 | 0 | 0 |
| SCN5A | NM_198056.2 | c.3236C>A | p.S1079Y | 0 | 0 | 0 | 0 | 0 | 0 | 0 | 5.0E-04 | 0 |
| SCN5A | NM_198056.2 | c.3338C>T | p.A1113V | 0 | 0 | 0 | 0 | 0 | 3.55E-04 | 0 | 0 | 0 |
| SCN5A | NM_198056.2 | c.3622G>A | p.E1208K | 2.7E-05 | 0 | 0 | 0 | 0 | 0 | 0 | 2.0E-04 | 0 |
| SCN5A | NM_198056.2 | c.3164A>G | p.D1055G | 0 | 0 | 0 | 0 | 0 | 0 | 0 | 0 | 0 |
| SCN5A | NM_198056.2 | c.3171_3172delTGinsA | CAGAC^1056ACAGAtgACCAAGAAGA | 0 | 0 | 0 | 0 | 0 | 0 | 0 | 0 | 0 |
| SCN5A | NM_198056.2 | c.3175C>T | p.Q1059* | 0 | 0 | 0 | 0 | 0 | 0 | 0 | 0 | 0 |
| SCN5A | NM_198056.2 | c.3207_3211dupGGAGG | GGCACG^1070GAGGggaggAGGAGTCCAG | 0 | 0 | 0 | 0 | 0 | 0 | 0 | 0 | 0 |
| SCN5A | NM_198056.2 | c.3228+2delT | NA | 0 | 0 | 0 | 0 | 0 | 0 | 0 | 0 | 0 |
| SCN5A | NM_198056.2 | c.3258_3261delAGAG | CCGGT^1085GGCCCagagGCCCCTCCGG | 0 | 0 | 0 | 0 | 0 | 0 | 0 | 0 | 0 |
| SCN5A | NM_198056.2 | c.327C>A | p.N109K | 8.95E-06 | 0 | 0 | 0 | 0 | 0 | 0 | 0 | 0 |
| SCN5A | NM_198056.2 | c.3284G>A | p.W1095* | 0 | 0 | 0 | 0 | 0 | 0 | 0 | 0 | 0 |
| SCN5A | NM_198056.2 | c.3345G>A | p.W1115* | 0 | 0 | 0 | 0 | 0 | 0 | 0 | 0 | 0 |
| SCN5A | NM_198056.2 | c.3352C>T | p.Q1118* | 0 | 0 | 0 | 0 | 0 | 0 | 0 | 0 | 0 |
| SCN5A | NM_198056.2 | c.3391-1G>A | NA | 0 | 0 | 0 | 0 | 0 | 0 | 0 | 0 | 0 |
| SCN5A | NM_198056.2 | c.3419G>C | p.S1140T | 0 | 0 | 0 | 0 | 0 | 0 | 0 | 0 | 0 |
| SCN5A | NM_198056.2 | c.3454G>T | p.E1152* | 0 | 0 | 0 | 0 | 0 | 0 | 0 | 0 | 0 |
| SCN5A | NM_198056.2 | c.3480delT | TCGGC^1159CAGGAtGTCAAGGACC | 0 | 0 | 0 | 0 | 0 | 0 | 0 | 0 | 0 |
| SCN5A | NM_198056.2 | c.3553_3554delCA | GGACACC^1184ACAcaGGCCCCAGGG | 0 | 0 | 0 | 0 | 0 | 0 | 0 | 0 | 0 |
| SCN5A | NM_198056.2 | c.3573G>A | p.W1191* | 0 | 0 | 0 | 0 | 0 | 0 | 0 | 0 | 0 |
| SCN5A | NM_198056.2 | c.3576G>A | p.W1192* | 0 | 0 | 0 | 0 | 0 | 0 | 0 | 0 | 0 |
| SCN5A | NM_198056.2 | c.361C>T | p.R121W | 0 | 0 | 0 | 0 | 0 | 0 | 0 | 0 | 0 |
| SCN5A | NM_198056.2 | c.362G>A | p.R121Q | 0 | 0 | 2.98E-05 | 0 | 0 | 0 | 3.25E-05 | 0 | 0 |
| SCN5A | NM_198056.2 | c.3653G>T | p.S1218I | 0 | 0 | 0 | 0 | 0 | 0 | 0 | 0 | 0 |
| SCN5A | NM_198056.2 | c.3656G>A | p.S1219N | 0 | 0 | 0 | 0 | 0 | 0 | 0 | 0 | 0 |
| SCN5A | NM_198056.2 | c.3666+1delG | NA | 0 | 0 | 0 | 0 | 0 | 0 | 0 | 0 | 0 |
| SCN5A | NM_198056.2 | c.3666+1G>A | NA | 0 | 0 | 0 | 0 | 0 | 0 | 0 | 0 | 0 |
| SCN5A | NM_198056.2 | c.3667delG | TTGTCTGCAG_I21E22_gCC^1224TTCGAGGA | 0 | 0 | 0 | 0 | 0 | 0 | 0 | 0 | 0 |
| SCN5A | NM_198056.2 | c.3673G>A | p.E1225K | 0 | 0 | 0 | 0 | 0 | 0 | 0 | 0 | 0 |
| SCN5A | NM_198056.2 | c.2674T>A | p.F892I | 0 | 0 | 0 | 0 | 0 | 0 | 0 | 0 | 0 |
| SCN5A | NM_198056.2 | c.3682T>C | p.Y1228H | 0 | 0 | 0 | 6.54E-05 | 0 | 0 | 0 | 0 | 0 |
| SCN5A | NM_198056.2 | c.3695G>A | p.R1232Q | 1.58E-05 | 0 | 2.91E-05 | 4.16E-05 | 0 | 0 | 9.75E-05 | 0 | 0 |
| SCN5A | NM_198056.2 | c.3708G>T | p.K1236N | 0 | 0 | 0 | 0 | 0 | 0 | 0 | 0 | 0 |
| SCN5A | NM_198056.2 | c.3716T>C | p.L1239P | 8.95E-06 | 0 | 0 | 0 | 0 | 0 | 0 | 0 | 0 |
| SCN5A | NM_198056.2 | c.371C>A | p.A124D | 0 | 0 | 0 | 0 | 0 | 0 | 0 | 0 | 0 |
| SCN5A | NM_198056.2 | c.3784G>A | p.G1262S | 1.6E-05 | 0 | 2.9E-05 | 0 | 0 | 5.3E-05 | 1.3E-04 | 0 | 0 |
| SCN5A | NM_198056.2 | c.3746T>A | p.V1249D | 0 | 0 | 0 | 0 | 0 | 0 | 0 | 0 | 0 |
| SCN5A | NM_198056.2 | c.3758A>G | p.E1253G | 0 | 0 | 0 | 0 | 0 | 0 | 0 | 0 | 0 |
| SCN5A | NM_198056.2 | c.376A>G | p.K126E | 0 | 0 | 0 | 0 | 0 | 0 | 0 | 0 | 0 |
| SCN5A | NM_198056.2 | c.3806A>G | p.N1269S | 8.95E-06 | 0 | 0 | 0 | 0 | 0 | 0 | 0 | 0 |
| SCN5A | NM_198056.2 | c.3813G>C | p.W1271C | 0 | 0 | 0 | 0 | 0 | 0 | 0 | 0 | 0 |
| SCN5A | NM_198056.2 | c.381dupT | TGTGAAG^127ATTtCTGGTTCACT | 0 | 0 | 0 | 0 | 0 | 0 | 0 | 0 | 0 |
| SCN5A | NM_198056.2 | c.3823G>A | p.D1275N | 8.95E-06 | 0 | 2.98E-05 | 0 | 0 | 0 | 0 | 0 | 0 |
| SCN5A | NM_198056.2 | c.3840+1G>A | NA | 8.96E-06 | 4.49E-05 | 0 | 0 | 0 | 0 | 0 | 0 | 0 |
| SCN5A | NM_198056.2 | c.3863C>G | p.A1288G | 0 | 0 | 0 | 0 | 0 | 0 | 0 | 0 | 0 |
| SCN5A | NM_198056.2 | c.3894delC | AGATG^1297GGCCCcATCAAGTCAC | 0 | 0 | 0 | 0 | 0 | 0 | 0 | 0 | 0 |
| SCN5A | NM_198056.2 | c.3932T>C | p.L1311P | 0 | 0 | 0 | 0 | 0 | 0 | 0 | 0 | 0 |
| SCN5A | NM_198056.2 | c.3940_3941delCT | TCTGAGA^1313GCTctGTCACGATTT | 0 | 0 | 0 | 0 | 0 | 0 | 0 | 0 | 0 |
| SCN5A | NM_198056.2 | c.3956G>T | p.G1319V | 7.39E-05 | 0 | 0 | 8.72E-05 | 0 | 0 | 0 | 0 | 0 |
| SCN5A | NM_198056.2 | c.3963+1G>A | NA | 0 | 0 | 0 | 0 | 0 | 0 | 0 | 0 | 0 |
| SCN5A | NM_198056.2 | c.3963+4A>G | NA | 0 | 0 | 0 | 0 | 0 | 0 | 3.52E-05 | 0 | 0 |
| SCN5A | NM_198056.2 | c.3968T>G | p.V1323G | 0 | 0 | 0 | 0 | 0 | 0 | 0 | 0 | 0 |
| SCN5A | NM_198056.2 | c.3995C>T | p.P1332Rfs | 0 | 0 | 0 | 0 | 0 | 0 | 0 | 0 | 0 |
| SCN5A | NM_198056.2 | c.3995delC | p.P1332L | 0 | 0 | 0 | 0 | 0 | 0 | 0 | 0 | 0 |
| SCN5A | NM_198056.2 | c.3G>A | p.M1I | 0 | 0 | 0 | 0 | 0 | 0 | 0 | 0 | 0 |
| SCN5A | NM_198056.2 | c.4030T>C | p.F1344L | 0 | 0 | 0 | 0 | 0 | 0 | 0 | 0 | 0 |
| SCN5A | NM_198056.2 | c.4031T>C | p.F1344S | 0 | 0 | 0 | 0 | 0 | 0 | 0 | 0 | 0 |
| SCN5A | NM_198056.2 | c.4035G>T | p.W1345C | 0 | 0 | 0 | 0 | 0 | 0 | 0 | 0 | 0 |
| SCN5A | NM_198056.2 | c.4036C>A | p.L1346I | 0 | 0 | 0 | 0 | 0 | 0 | 0 | 0 | 0 |
| SCN5A | NM_198056.2 | c.4037T>C | p.L1346P | 0 | 0 | 0 | 0 | 0 | 0 | 0 | 0 | 0 |
| SCN5A | NM_198056.2 | c.4049T>C | p.I1350T | 0 | 0 | 0 | 0 | 0 | 0 | 0 | 0 | 0 |
| SCN5A | NM_198056.2 | c.4052T>G | p.M1351R | 0 | 0 | 0 | 0 | 0 | 0 | 0 | 0 | 0 |
| SCN5A | NM_198056.2 | c.4057G>A | p.V1353M | 3.58E-05 | 0 | 2.98E-05 | 0 | 0 | 5.8E-05 | 3.25E-05 | 0 | 0 |
| SCN5A | NM_198056.2 | c.4067_4068delTT | GTGAAC^1355CTCTttGCGGGGAAGT | 0 | 0 | 0 | 0 | 0 | 0 | 0 | 0 | 0 |
| SCN5A | NM_198056.2 | c.4072G>T | p.G1358W | 0 | 0 | 0 | 0 | 0 | 0 | 0 | 0 | 0 |
| SCN5A | NM_198056.2 | c.4077G>T | p.K1359N | 0 | 0 | 0 | 0 | 0 | 0 | 0 | 0 | 0 |
| SCN5A | NM_198056.2 | c.4079T>G | p.F1360C | 0 | 0 | 0 | 0 | 0 | 0 | 0 | 0 | 0 |
| SCN5A | NM_198056.2 | c.407T>C | p.L136P | 0 | 0 | 0 | 0 | 0 | 0 | 0 | 0 | 0 |
| SCN5A | NM_198056.2 | c.4088G>A | p.C1363Y | 0 | 0 | 0 | 0 | 0 | 0 | 0 | 0 | 0 |
| SCN5A | NM_198056.2 | c.4094A>G | p.N1365S | 0 | 0 | 0 | 0 | 0 | 0 | 0 | 0 | 0 |
| SCN5A | NM_198056.2 | c.410_418dupTCATGTGCA | ATCATG^139TGCAtcatgtgcaCCATCCTCAC | 0 | 0 | 0 | 0 | 0 | 0 | 0 | 0 | 0 |
| SCN5A | NM_198056.2 | c.4118T>A | p.L1373* | 0 | 0 | 0 | 0 | 0 | 0 | 0 | 0 | 0 |
| SCN5A | NM_198056.2 | c.4132G>A | p.V1378M | 0 | 0 | 0 | 0 | 0 | 0 | 0 | 0 | 0 |
| SCN5A | NM_198056.2 | c.4140C>G | p.N1380K | 0 | 0 | 0 | 0 | 0 | 0 | 0 | 0 | 0 |
| SCN5A | NM_198056.2 | c.4145G>T | p.S1382I | 0 | 0 | 0 | 0 | 0 | 0 | 0 | 0 | 0 |
| SCN5A | NM_198056.2 | c.4147C>T | p.Q1383* | 0 | 0 | 0 | 0 | 0 | 0 | 0 | 0 | 0 |
| SCN5A | NM_198056.2 | c.4182C>G | p.Y1394* | 0 | 0 | 0 | 0 | 0 | 0 | 0 | 0 | 0 |
| SCN5A | NM_000335.4 | c.4187delA | p.K1396Rfs | 0 | 0 | 0 | 0 | 0 | 0 | 0 | 0 | 0 |
| SCN5A | NM_198056.2 | c.4213G>A | p.V1405L | 0 | 0 | 0 | 0 | 0 | 0 | 0 | 0 | 0 |
| SCN5A | NM_198056.2 | c.4213G>C | p.V1405M | 0 | 0 | 0 | 0 | 0 | 0 | 0 | 0 | 0 |
| SCN5A | NM_198056.2 | c.4216G>C | p.G1406R | 0 | 0 | 0 | 0 | 0 | 0 | 0 | 0 | 0 |
| SCN5A | NM_198056.2 | c.3682T>C | p.Y1228H | 0 | 0 | 0 | 6.54E-05 | 0 | 0 | 0 | 0 | 0 |
| SCN5A | NM_198056.2 | c.4217G>A | p.G1406E | 0 | 0 | 0 | 0 | 0 | 0 | 0 | 0 | 0 |
| SCN5A | NM_198056.2 | c.4222G>A | p.G1408R | 0 | 0 | 0 | 0 | 0 | 0 | 0 | 0 | 0 |
| SCN5A | NM_198056.2 | c.4226A>G | p.Y1409C | 0 | 0 | 0 | 0 | 0 | 0 | 0 | 0 | 0 |
| SCN5A | NM_198056.2 | c.4227C>G | p.Y1409* | 0 | 0 | 0 | 0 | 0 | 0 | 0 | 0 | 0 |
| SCN5A | NM_198056.2 | c.4234C>T | p.L1412F | 0 | 0 | 0 | 0 | 0 | 0 | 0 | 0 | 0 |
| SCN5A | NM_198056.2 | c.4255A>G | p.K1419E | 0 | 0 | 0 | 0 | 0 | 0 | 0 | 0 | 0 |
| SCN5A | NM_198056.2 | c.4258G>C | p.G1420R | 0 | 0 | 0 | 0 | 0 | 0 | 0 | 0 | 0 |
| SCN5A | NM_000335.4 | c.4259G>A | p.W1420* | 0 | 0 | 0 | 0 | 0 | 0 | 0 | 0 | 0 |
| SCN5A | NM_198056.2 | c.4279G>T | p.A1427S | 0 | 0 | 0 | 0 | 0 | 0 | 0 | 0 | 0 |
| SCN5A | NM_198056.2 | c.4283C>T | p.A1428V | 0 | 0 | 0 | 0 | 0 | 0 | 0 | 0 | 0 |
| SCN5A | NM_198056.2 | c.4288G>A | p.D1430N | 0 | 0 | 0 | 0 | 0 | 0 | 0 | 0 | 0 |
| SCN5A | NM_198056.2 | c.4294A>G | p.R1432G | 0 | 0 | 0 | 0 | 0 | 0 | 0 | 0 | 0 |
| SCN5A | NM_198056.2 | c.4298G>T | p.G1433V | 0 | 0 | 0 | 0 | 0 | 0 | 0 | 0 | 0 |
| SCN5A | NM_198056.2 | c.4299+1dupG | NA | 0 | 0 | 0 | 0 | 0 | 0 | 0 | 0 | 0 |
| SCN5A | NM_198056.2 | c.4299+1G>T | NA | 0 | 0 | 0 | 0 | 0 | 0 | 0 | 0 | 0 |
| SCN5A | NM_198056.2 | c.4299G>A | IVS25 ds G-A -1 | 0 | 0 | 0 | 0 | 0 | 0 | 0 | 0 | 0 |
| SCN5A | NM_198056.2 | c.4300-1G>A | NA | 0 | 0 | 0 | 0 | 0 | 0 | 0 | 0 | 0 |
| SCN5A | NM_198056.2 | c.4302T>G | p.Y1434* | 0 | 0 | 0 | 0 | 0 | 0 | 0 | 0 | 0 |
| SCN5A | NM_198056.2 | c.4313C>T | p.P1438L | 0 | 0 | 0 | 0 | 0 | 0 | 0 | 0 | 0 |
| SCN5A | NM_198056.2 | c.4320G>A | p.W1440* | 0 | 0 | 0 | 0 | 0 | 0 | 0 | 0 | 0 |
| SCN5A | NM_198056.2 | c.4321G>C | p.E1441Q | 0 | 0 | 0 | 0 | 0 | 0 | 0 | 0 | 0 |
| SCN5A | NM_198056.2 | c.4328A>G | p.N1443S | 0 | 0 | 2.98E-04 | 0 | 0 | 0 | 0 | 0 | 0 |
| SCN5A | NM_198056.2 | c.4534C>T | p.R1512W | 1.8E-05 | 0 | 2.68E-04 | 0 | 0 | 0 | 0 | 0 | 0 |
| SCN5A | NM_198056.2 | c.4810G>A | p.V1604M | 2.4E-05 | 0 | 0 | 1.25E-04 | 0 | 5.3E-05 | 3.3E-05 | 0 | 0 |
| SCN5A | NM_198056.2 | c.5297T>C | p.M1766T | 0 | 0 | 0 | 0 | 0 | 0 | 1.3E-04 | 0 | 0 |
| SCN5A | NM_198056.2 | c.6046G>A | p.V2016M | 1.0E-05 | 0 | 0 | 1.3E-04 | 0 | 0 | 2.55E-04 | 0 | 0 |
| SCN5A | NM_198056.2 | c.4342A>C | p.I1448L | 5.38E-05 | 4.49E-05 | 0 | 0 | 0 | 0 | 0 | 0 | 0 |
| SCN5A | NM_198056.2 | c.4343T>C | p.I1448T | 0 | 0 | 0 | 0 | 0 | 0 | 0 | 0 | 0 |
| SCN5A | NM_198056.2 | c.4346A>C | p.Y1449C | 0 | 0 | 0 | 0 | 0 | 0 | 0 | 0 | 0 |
| SCN5A | NM_198056.2 | c.4346A>G | p.Y1449S | 0 | 0 | 0 | 0 | 0 | 0 | 0 | 0 | 0 |
| SCN5A | NM_198056.2 | c.4352T>A | p.V1451D | 0 | 0 | 0 | 0 | 0 | 0 | 0 | 0 | 0 |
| SCN5A | NM_198056.2 | c.4376_4379delTCTT | TTTGGG^1458TCTTtcttCACCCTGAAC | 0 | 0 | 0 | 0 | 0 | 0 | 0 | 0 | 0 |
| SCN5A | NM_198056.2 | c.4387A>T | p.N1463Y | 0 | 0 | 0 | 0 | 0 | 0 | 0 | 0 | 0 |
| SCN5A | NM_198056.2 | c.4389_4396delCCTCTTTA | TCACC^1462CTGAAcctctttaTTGGTGTCAT | 0 | 0 | 0 | 0 | 0 | 0 | 0 | 0 | 0 |
| SCN5A | NM_198056.2 | c.4402_4405delGTCA | CTTTATT^1467GGTgtcaTCATTGACAA | 0 | 0 | 0 | 0 | 0 | 0 | 0 | 0 | 0 |
| SCN5A | NM_198056.2 | c.4402G>T | p.V1468F | 0 | 0 | 0 | 0 | 0 | 0 | 0 | 0 | 0 |
| SCN5A | NM_198056.2 | c.4426C>T | p.Q1476* | 0 | 0 | 0 | 0 | 0 | 0 | 0 | 0 | 0 |
| SCN5A | NM_198056.2 | c.4436_4437+1delAGA | NA | 0 | 0 | 0 | 0 | 0 | 0 | 0 | 0 | 0 |
| SCN5A | NM_198056.2 | c.4438-1C>T | NA | 0 | 0 | 0 | 0 | 0 | 0 | 0 | 0 | 0 |
| SCN5A | NM_198056.2 | c.4453A>G | p.I1485V | 0 | 0 | 0 | 0 | 0 | 0 | 0 | 0 | 0 |
| SCN5A | NM_198056.2 | c.4477_4479delAAG | GGAGCAG^1492AAGaagTACTACAATG | 0 | 0 | 0 | 0 | 0 | 0 | 0 | 0 | 0 |
| SCN5A | NM_198056.2 | c.4477A>T | p.K1493* | 0 | 0 | 0 | 0 | 0 | 0 | 0 | 0 | 0 |
| SCN5A | NM_198056.2 | c.4480T>A | p.Y1494N | 0 | 0 | 0 | 0 | 0 | 0 | 0 | 0 | 0 |
| SCN5A | NM_198056.2 | c.4504G>A | p.G1502S | 0 | 0 | 0 | 0 | 0 | 0 | 3.25E-05 | 0 | 0 |
| SCN5A | NM_198056.2 | c.4516C>A | p.P1506S | 0 | 0 | 0 | 0 | 0 | 0 | 0 | 0 | 0 |
| SCN5A | NM_198056.2 | c.4516C>T | p.P1506T | 0 | 0 | 0 | 0 | 0 | 0 | 0 | 0 | 0 |
| SCN5A | NM_198056.2 | c.4562T>A | p.I1521K | 0 | 0 | 0 | 0 | 0 | 0 | 0 | 0 | 0 |
| SCN5A | NM_198056.2 | c.4642G>A | p.E1548K | 6.66E-05 | 0 | 0 | 0 | 0 | 0 | 0 | 0 | 0 |
| SCN5A | NM_198056.2 | c.4657A>C | p.S1553R | 0 | 0 | 0 | 0 | 0 | 0 | 0 | 0 | 0 |
| SCN5A | NM_198056.2 | c.4708_4710dupATC | TGTGGCC^1570ATCatcTTCACAGGCG | 0 | 0 | 0 | 0 | 0 | 0 | 0 | 0 | 0 |
| SCN5A | NM_198056.2 | c.4712T>G | p.F1571C | 0 | 0 | 0 | 0 | 0 | 0 | 0 | 0 | 0 |
| SCN5A | NM_198056.2 | c.4719C>T | IVS28b ds C-T -95 | 1.58E-05 | 0 | 2.91E-05 | 0.000229 | 0 | 0 | 0 | 0 | 0 |
| SCN5A | NM_198056.2 | c.4720G>A | p.E1574K | 0 | 0 | 0 | 0 | 0 | 0 | 0 | 0 | 0 |
| SCN5A | NM_198056.2 | c.4732_4733dupAA | GTATT^1577GTCAAaaGCTGGCTGCC | 0 | 0 | 0 | 0 | 0 | 0 | 0 | 0 | 0 |
| SCN5A | NM_198056.2 | c.4745T>C | p.L1582P | 0 | 0 | 0 | 0 | 0 | 0 | 0 | 0 | 0 |
| SCN5A | NM_198056.2 | c.4217G>A | p.G1406E | 0 | 0 | 0 | 0 | 0 | 0 | 0 | 0 | 0 |
| SCN5A | NM_198056.2 | c.4747C>T | p.R1583C | 1.79E-05 | 0 | 0 | 0 | 0 | 0 | 0 | 0 | 0 |
| SCN5A | NM_198056.2 | c.4748G>A | p.R1583H | 1.79E-05 | 0 | 2.98E-05 | 0 | 0 | 5.80E-05 | 0 | 0 | 0 |
| SCN5A | NM_198056.2 | c.4772G>A | p.W1591* | 0 | 0 | 0 | 0 | 0 | 0 | 0 | 0 | 0 |
| SCN5A | NM_198056.2 | c.4773G>A | p.W1591* | 0 | 0 | 0 | 0 | 0 | 0 | 0 | 0 | 0 |
| SCN5A | NM_198056.2 | c.477T>A | p.Y159* | 0 | 0 | 0 | 0 | 0 | 0 | 0 | 0 | 0 |
| SCN5A | NM_198056.2 | c.4813+3_4813+6dupGGGT | NA | 0 | 0 | 0 | 0 | 0 | 0 | 0 | 0 | 0 |
| SCN5A | NM_198056.2 | c.481G>C | p.E161Q | 0 | 0 | 0 | 0 | 0 | 0 | 0 | 0 | 0 |
| SCN5A | NM_198056.2 | c.483-10_492del20 | NA | 0 | 0 | 0 | 0 | 0 | 0 | 0 | 0 | 0 |
| SCN5A | NM_198056.2 | c.4838A>T | p.Q1613L | 0 | 0 | 0 | 0 | 0 | 0 | 0 | 0 | 0 |
| SCN5A | NM_198056.2 | c.4845_4847delCTTinsGTA | p.Y1615* | 0 | 0 | 0 | 0 | 0 | 0 | 0 | 0 | 0 |
| SCN5A | NM_198056.2 | c.4845C>A | p.Y1615* | 0 | 0 | 0 | 0 | 0 | 0 | 0 | 0 | 0 |
| SCN5A | NM_198056.2 | c.4856delC | TTCTTC^1618TCCCcGACGCTCTTC | 0 | 0 | 0 | 0 | 0 | 0 | 0 | 0 | 0 |
| SCN5A | NM_198056.2 | c.4867delC | GACGCTC^1622TTCcGAGTCATCCG | 0 | 0 | 0 | 0 | 0 | 0 | 0 | 0 | 0 |
| SCN5A | NM_198056.2 | c.486delC | TGCCCAG_I4E5_GTAc^163ACCTTCACCG | 0 | 0 | 0 | 0 | 0 | 0 | 0 | 0 | 0 |
| SCN5A | NM_198056.2 | c.4885C>G | p.R1629G | 0 | 0 | 0 | 0 | 0 | 0 | 0 | 0 | 0 |
| SCN5A | NM_198056.2 | c.4885C>T | p.R1629* | 8.95E-06 | 0 | 0 | 0 | 0 | 0 | 0 | 0 | 0 |
| SCN5A | NM_198056.2 | c.4886G>A | p.R1629Q | 0 | 0 | 5.96E-05 | 0 | 0 | 0 | 3.25E-05 | 0 | 0 |
| SCN5A | NM_198056.2 | c.4912C>T | p.R1638* | 2.69E-05 | 0 | 0 | 0 | 0 | 0 | 0 | 0 | 0 |
| SCN5A | NM_198056.2 | c.4925G>A | p.G1642E | 0 | 0 | 0 | 0 | 0 | 0 | 0 | 0 | 0 |
| SCN5A | NM_198056.2 | c.4930C>T | p.R1644C | 0 | 0 | 0 | 0 | 0 | 0 | 3.25E-05 | 0 | 0 |
| SCN5A | NM_198056.2 | c.4946C>T | p.A1649V | 0 | 0 | 0 | 0 | 0 | 0 | 0 | 0 | 0 |
| SCN5A | NM_198056.2 | c.4952dupT | TTGCC^1650CTCATtGATGTCCCTG | 0 | 0 | 0 | 0 | 0 | 0 | 0 | 0 | 0 |
| SCN5A | NM_198056.2 | c.4981G>A | p.G1661R | 0 | 0 | 0 | 0 | 0 | 0 | 0 | 0 | 0 |
| SCN5A | NM_198056.2 | c.4981G>C | p.G1661R | 0 | 0 | 0 | 0 | 0 | 0 | 0 | 0 | 0 |
| SCN5A | NM_198056.2 | c.4G>A | p.A2T | 0 | 0 | 0 | 0 | 0 | 0 | 0 | 0 | 0 |
| SCN5A | NM_198056.2 | c.5015C>A | p.S1672Y | 0 | 0 | 0 | 0 | 0 | 0 | 0 | 0 | 0 |
| SCN5A | NM_198056.2 | c.5040_5042delTTAinsC | CCAAC^1679TTCGCttaTGTCAAGTGG | 0 | 0 | 0 | 0 | 0 | 0 | 0 | 0 | 0 |
| SCN5A | NM_198056.2 | c.5068_5069delGA | TGGCATC^1689GACgaCATGTTCAAC | 0 | 0 | 0 | 0 | 0 | 0 | 0 | 0 | 0 |
| SCN5A | NM_198056.2 | c.5068G>A | p.D1690N | 0 | 0 | 2.98E-05 | 0 | 0 | 0 | 0 | 0 | 0 |
| SCN5A | NM_198056.2 | c.5092G>A | p.A1698T | 8.95E-06 | 0 | 5.96E-05 | 0 | 0 | 0 | 0 | 0 | 0 |
| SCN5A | NM_198056.2 | c.5118G>C | p.Q1706H | 0 | 0 | 0 | 0 | 0 | 0 | 0 | 0 | 0 |
| SCN5A | NM_198056.2 | c.5123C>A | p.T1708N | 0 | 0 | 0 | 0 | 0 | 0 | 0 | 0 | 0 |
| SCN5A | NM_198056.2 | c.5124_5126delCAC | TCCAG^1707ATCACcacGTCGGCCGGC | 0 | 0 | 0 | 0 | 0 | 0 | 0 | 0 | 0 |
| SCN5A | NM_198056.2 | c.5126C>G | p.T1709R | 0 | 0 | 0 | 0 | 0 | 0 | 0 | 0 | 0 |
| SCN5A | NM_198056.2 | c.5126C>T | p.T1709M | 8.95E-06 | 0 | 0 | 0 | 0 | 0 | 0 | 0 | 0 |
| SCN5A | NM_198056.2 | c.5134G>A | p.G1712S | 0 | 0 | 2.98E-05 | 0 | 0 | 0 | 0 | 0 | 0 |
| SCN5A | NM_198056.2 | c.5141A>G | p.D1714G | 0 | 0 | 0 | 0 | 0 | 0 | 0 | 0 | 0 |
| SCN5A | NM_198056.2 | c.5150T>C | p.L1717P | 0 | 0 | 0 | 0 | 0 | 0 | 0 | 0 | 0 |
| SCN5A | NM_198056.2 | c.5157delC | CTCCTC^1718AGCCcCATCCTCAAC | 0 | 0 | 0 | 0 | 0 | 0 | 0 | 0 | 0 |
| SCN5A | NM_198056.2 | c.5164A>G | p.N1722D | 0 | 0 | 0 | 0 | 0 | 0 | 0 | 0 | 0 |
| SCN5A | NM_198056.2 | c.5182T>C | p.C1728R | 0 | 0 | 0 | 0 | 0 | 0 | 0 | 0 | 0 |
| SCN5A | NM_198056.2 | c.5184C>G | p.C1728W | 0 | 0 | 0 | 0 | 0 | 0 | 0 | 0 | 0 |
| SCN5A | NM_198056.2 | c.5218G>A | p.G1740R | 0 | 0 | 0 | 0 | 0 | 0 | 0 | 0 | 0 |
| SCN5A | NM_198056.2 | c.5227G>A | p.G1743R | 0 | 0 | 0 | 0 | 0 | 0 | 0 | 0 | 0 |
| SCN5A | NM_198056.2 | c.5228G>A | p.G1743E | 0 | 0 | 0 | 0 | 0 | 0 | 0 | 0 | 0 |
| SCN5A | NM_198056.2 | c.5243G>A | p.G1748D | 0 | 0 | 0 | 0 | 0 | 0 | 0 | 0 | 0 |
| SCN5A | NM_198056.2 | c.525G>C | p.K175N | 0 | 0 | 0 | 0 | 0 | 0 | 0 | 0 | 0 |
| SCN5A | NM_198056.2 | c.5290delG | CCTCATC^1763GTGgTCAACATGTA | 0 | 0 | 0 | 0 | 0 | 0 | 0 | 0 | 0 |
| SCN5A | NM_198056.2 | c.5290G>T | p.V1764F | 0 | 0 | 0 | 0 | 0 | 0 | 0 | 0 | 0 |
| SCN5A | NM_198056.2 | c.5321A>G | p.N1774S | 0 | 0 | 0 | 0 | 0 | 0 | 0 | 0 | 0 |
| SCN5A | NM_198056.2 | c.5324delT | CTGGAG^1774AACTtCAGCGTGGCC | 0 | 0 | 0 | 0 | 0 | 0 | 0 | 0 | 0 |
| SCN5A | NM_198056.2 | c.533C>G | c.533C>G | 0 | 0 | 0 | 0 | 0 | 0 | 0 | 0 | 0 |
| SCN5A | NM_198056.2 | c.5350G>A | p.E1784K | 0 | 0 | 0 | 0 | 0 | 0 | 0 | 0 | 0 |
| SCN5A | NM_198056.2 | c.5356_5357delCT | CACCGAG^1785CCCctGAGTGAGGAC | 0 | 0 | 0 | 0 | 0 | 0 | 0 | 0 | 0 |
| SCN5A | NM_198056.2 | c.5357T>A | p.L1786Q | 0 | 0 | 0 | 0 | 0 | 0 | 0 | 0 | 0 |
| SCN5A | NM_198056.2 | c.535C>T | p.R179* | 0 | 0 | 2.98E-05 | 0 | 0 | 0 | 0 | 0 | 0 |
| SCN5A | NM_000335.4 | c.5382_5384dupTGA | p.Y1794_E1795insD | 0 | 0 | 0 | 0 | 0 | 0 | 0 | 0 | 0 |
| SCN5A | NM_198056.2 | c.5383T>C | p.Y1795H | 0 | 0 | 0 | 0 | 0 | 0 | 0 | 0 | 0 |
| SCN5A | NM_198056.2 | c.5435C>A | p.S1812* | 0 | 0 | 0 | 0 | 0 | 0 | 0 | 0 | 0 |
| SCN5A | NM_198056.2 | c.5445dupT | GGTCCTG^1815TCTtGACTTTGCCG | 0 | 0 | 0 | 0 | 0 | 0 | 0 | 0 | 0 |
| SCN5A | NM_198056.2 | c.544T>C | p.C182R | 0 | 0 | 0 | 0 | 0 | 0 | 0 | 0 | 0 |
| SCN5A | NM_198056.2 | c.5540G>A | p.R1847H | 0 | 0 | 0 | 8.33E-05 | 9.85E-05 | 0 | 0 | 0 | 0 |
| SCN5A | NM_198056.2 | c.5549G>C | p.C1850S | 0 | 0 | 0 | 0 | 0 | 0 | 0 | 0 | 0 |
| SCN5A | NM_198056.2 | c.554C>T | p.A185V | 3.61E-05 | 0 | 0 | 0 | 0 | 0 | 0 | 0 | 0 |
| SCN5A | NM_198056.2 | c.5578dupA | TTCACC^1859AAAAaGGGTCCTGGG | 0 | 0 | 0 | 0 | 0 | 0 | 0 | 0 | 0 |
| SCN5A | NM_198056.2 | c.5581G>A | p.V1861I | 0 | 0 | 0 | 0 | 0 | 0 | 0 | 0 | 0 |
| SCN5A | NM_198056.2 | c.560C>T | p.T187I | 0 | 0 | 0 | 0 | 0 | 0 | 0 | 0 | 0 |
| SCN5A | NM_198056.2 | c.5616G>C | p.K1872N | 0 | 0 | 0 | 0 | 0 | 0 | 0 | 0 | 0 |
| SCN5A | NM_198056.2 | c.5623_5625dupATG | p.M1875dup | 0 | 0 | 0 | 0 | 0 | 0.000106 | 0 | 0 | 0 |
| SCN5A | NM_198056.2 | c.5692C>T | p.R1898C | 6.31E-05 | 0 | 0 | 4.16E-05 | 0 | 5.30E-05 | 0 | 0 | 0 |
| SCN5A | NM_198056.2 | c.579G>A | p.W193* | 0 | 0 | 0 | 0 | 0 | 0 | 0 | 0 | 0 |
| SCN5A | NM_198056.2 | c.5803G>A | p.G1935S | 8.97E-06 | 0 | 5.96E-05 | 0 | 0 | 5.80E-05 | 9.75E-05 | 0 | 0 |
| SCN5A | NM_198056.2 | c.5812G>A | p.E1938K | 0 | 0 | 5.82E-05 | 4.17E-05 | 0 | 5.31E-05 | 0 | 0 | 0 |
| SCN5A | NM_198056.2 | c.5845G>C | p.A1949P | 0 | 0 | 0 | 0 | 0 | 0 | 0 | 0 | 0 |
| SCN5A | NM_000335.4 | c.611+3_611+4dupAA | NA | 0 | 0 | 0 | 0 | 0 | 0 | 0 | 0 | 0 |
| SCN5A | NM_198056.2 | c.611C>T | p.A204V | 0 | 0 | 0 | 0 | 0 | 0 | 0 | 0 | 0 |
| SCN5A | NM_198056.2 | c.612-2A>G | NA | 0 | 0 | 0 | 0 | 0 | 0 | 0 | 0 | 0 |
| SCN5A | NM_198056.2 | c.635T>A | p.L212Q | 0 | 0 | 0 | 0 | 0 | 0 | 0 | 0 | 0 |
| SCN5A | NM_198056.2 | c.656_657insATTCA | CAGCC^218TTACGattcaCACCTTCCGA | 9.14E-06 | 0 | 0 | 0 | 0 | 0 | 0 | 0 | 0 |
| SCN5A | NM_198056.2 | c.656G>A | p.R219H | 0 | 0 | 0 | 0 | 0 | 0 | 0 | 0 | 0 |
| SCN5A | NM_198056.2 | c.667G>C | p.V223L | 0 | 0 | 0 | 0 | 0 | 0 | 0 | 0 | 0 |
| SCN5A | NM_198056.2 | c.692_693delCA | AAAACT^230ATATcaGTCATTTCAG | 0 | 0 | 0 | 0 | 0 | 0 | 0 | 0 | 0 |
| SCN5A | NM_198056.2 | c.745A>T | p.K249* | 0 | 0 | 0 | 0 | 0 | 0 | 0 | 0 | 0 |
| SCN5A | NM_198056.2 | c.784A>C | p.S262R | 0 | 0 | 0 | 0 | 0 | 0 | 0 | 0 | 0 |
| SCN5A | NM_198056.2 | c.808C>A | p.Q270K | 0 | 0 | 0 | 0 | 0 | 0 | 0 | 0 | 0 |
| SCN5A | NM_198056.2 | c.827T>A | p.L276Q | 0 | 0 | 0 | 0 | 0 | 0 | 0 | 0 | 0 |
| SCN5A | NM_198056.2 | c.827T>C | p.L276P | 0 | 0 | 0 | 0 | 0 | 0 | 0 | 0 | 0 |
| SCN5A | NM_198056.2 | c.832C>G | p.H278D | 0 | 0 | 0 | 0 | 0 | 0 | 0 | 0 | 0 |
| SCN5A | NM_198056.2 | c.839G>A | p.C280Y | 0 | 0 | 0 | 0 | 0 | 0 | 0 | 0 | 0 |
| SCN5A | NM_198056.2 | c.841G>A | p.V281M | 8.95E-06 | 0 | 0 | 0 | 0 | 5.8E-05 | 0 | 0 | 0 |
| SCN5A | NM_198056.2 | c.844C>T | p.R282C | 0 | 0 | 0 | 0 | 0 | 0 | 0 | 0 | 0 |
| SCN5A | NM_198056.2 | c.845G>A | p.R282H | 1.79E-05 | 0 | 0 | 0 | 0 | 5.8E-05 | 0 | 0 | 0 |
| SCN5A | NM_198056.2 | c.880G>A | p.V294M | 3.16E-05 | 0 | 0 | 0 | 9.86E-05 | 0 | 0 | 0 | 0 |
| SCN5A | NM_198056.2 | c.898G>A | p.V300I | 8.99E-06 | 0 | 0 | 0 | 0 | 0 | 0 | 0 | 0 |
| SCN5A | NM_198056.2 | c.903G>A | p.W301* | 0 | 0 | 0 | 0 | 0 | 0 | 0 | 0 | 0 |
| SCN5A | NM_198056.2 | c.934+1G>A | NA | 0 | 0 | 0 | 0 | 0 | 0 | 0 | 0 | 0 |
| SCN5A | NM_198056.2 | c.944T>C | p.L315P | 0 | 0 | 0 | 0 | 0 | 0 | 0 | 0 | 0 |
| SCN5A | NM_198056.2 | c.951G>C | p.K317N | 0 | 0 | 0 | 0 | 0 | 0 | 0 | 0 | 0 |
| SCN5A | NM_198056.2 | c.974T>G | p.L325R | 0 | 0 | 0 | 0 | 0 | 0 | 0 | 0 | 0 |
| SCN5A | NM_198056.2 | c.5321A>G | p.N1774S | 0 | 0 | 0 | 0 | 0 | 0 | 0 | 0 | 0 |
| SCN5A | NM_198056.2 | c.5324delT | CTGGAG^1774AACTtCAGCGTGGCC | 0 | 0 | 0 | 0 | 0 | 0 | 0 | 0 | 0 |
| SCN5A | NM_198056.2 | c.998+1G>A | NA | 8.96E-06 | 0 | 0 | 0 | 0 | 0 | 0 | 0 | 0 |
| TRPM4 | NM_017636.3 | c.1663G>A | p.G555R | 0 | 0 | 0 | 0 | 0 | 0 | 0 | 0 | 0 |
| TRPM4 | NM_017636.3 | c.2317T>A | p.F773I | 0 | 0 | 0 | 0 | 0 | 0 | 0 | 0 | 0 |
| TRPM4 | NM_017636.3 | c.2336C>G | p.P779R | 0 | 0 | 0 | 0 | 0 | 0 | 0 | 0 | 0 |
| TRPM4 | NM_017636.3 | c.2618C>T | p.T873I | 0 | 0 | 0 | 0 | 0 | 0 | 0 | 0 | 0 |
| TRPM4 | NM_017636.3 | c.3224T>C | p.L1075P | 2.37E-04 | 3.9E-05 | 0 | 8.3E-05 | 0 | 0 | 0 | 0 | 0 |
| TTN | NM_133378.4 | c.58991T>A | p.V19664E | 0 | 0 | 0 | 0 | 0 | 0 | 0 | 0 | 0 |
| TTN | NM_133378.4 | c.94353delT | TGGCT^31450GAAACtAACCAACAGA | 0 | 0 | 0 | 0 | 0 | 0 | 0 | 0 | 0 |

AA# = African American; AMR = American; AFR = African; ΔAA = aminoacid change; ΔNA = nucleotide change; EAS = East Asian; FIN = Finnish; JEW = Ashkenazi Jewish; 3KJPNv2 = Japan Biobank; NA = not available; NFE = non-Finnish European; SAS = South Asian; TWB = Taiwan Biobank.

Supplementary Table 2. Several variants have allele frequency more than 0.001 in at least one ancestry according to 1000 Genome Project (1000G) or Exome Sequencing Project (ESP) but were reclassified as rare variants by the [Genome Aggregation Database](http://gnomad.broadinstitute.org/about) (gnomAD# version 2.0, the currently largest reference database).

| Gene | Transcript | ΔNA(Ref) | ΔAA | 1000G | | | | | ESP | | gnomAD | | | | |
| --- | --- | --- | --- | --- | --- | --- | --- | --- | --- | --- | --- | --- | --- | --- | --- |
|  |  |  |  | EUR | AMR | AFR | EAS | SAS | AA | EA# | NFE | AMR | AFR | EAS | SAS |
| CACNA1C | NM_000719.6 | 898A>G | N300D | 0 | 0 | 0 | 0 | 0 | 0.002* | 0.003* | 0.000045 | 0.00006 | 0 | 0 | 0 |
| SCN5A | NM_198056.2 | 1652C>T | A551V | 0 | 0.0014* | 0 | 0 | 0 | 0.0002 | 0.0001 | 0.000055 | 0.000087 | 0.000042 | 0.000053 | 0 |
| SCN5A | NM_198056.2 | 1940C>A | A647D | 0 | 0 | 0 | 0.001* | 0 | 0 | 0 | 0 | 0 | 0 | 0 | 0 |
| SCN5A | NM_198056.2 | 2236G>A | E746K | 0.001* | 0 | 0.0008 | 0 | 0 | 0 | 0 | 0.000016 | 0.000058 | 0.000083 | 0 | 0 |
| SCN5A | NM_198056.2 | 5692C>T | R1898C | 0 | 0 | 0 | 0.002* | 0 | 0 | 0.0001 | 0.000063 | 0 | 0.000042 | 0.000053 | 0 |

AA = African American; AFR = African; AMR = American; ΔAA = aminoacid change; ΔNA = nucleotide change; EAS = East Asian; EA# = European American; EUR = European; NFE = non-Finnish European; Ref = reference; SAS = South Asian. * >0.001

$gnomAD: The data set provided on this website spans 123,136 exome sequences and 15,496 whole-genome sequences from unrelated individuals sequenced as part of various disease-specific and population genetic studies

**Supplementary reference**

Beziau, D.M., Barc, J., O'Hara, T., Le Gloan, L., Amarouch, M.Y., Solnon, A., et al. (2014). Complex Brugada syndrome inheritance in a family harbouring compound SCN5A and CACNA1C mutations. *Basic Res Cardiol* 109(6)**,** 446. doi: 10.1007/s00395-014-0446-5.

Kapplinger, J.D., Tester, D.J., Alders, M., Benito, B., Berthet, M., Brugada, J., et al. (2010). An international compendium of mutations in the SCN5A-encoded cardiac sodium channel in patients referred for Brugada syndrome genetic testing. *Heart Rhythm* 7(1)**,** 33-46. doi: 10.1016/j.hrthm.2009.09.069.

Selga, E., Campuzano, O., Pinsach-Abuin, M.L., Perez-Serra, A., Mademont-Soler, I., Riuro, H., et al. (2015). Comprehensive Genetic Characterization of a Spanish Brugada Syndrome Cohort. *PLoS One* 10(7)**,** e0132888. doi: 10.1371/journal.pone.0132888.
